# Targeted Mechanogenetics to Modulate Specific Deep Neural Circuits

**DOI:** 10.1002/advs.78177

**Published:** 2026-10-06

**Authors:** Xuandi Hou, Jianing Jing, Zhuohan Shi, Lei Sun

**Affiliations:** ^1^ Department of Biomedical Engineering The Hong Kong Polytechnic University Hung Hom Hong Kong SAR China

**Keywords:** mechanogenetics, nanotechnology, neural circuits, transcranial ultrasound stimulation

## Abstract

Sonogenetics utilizes ultrasound for targeted, cell‐type‐specific modulation of neuronal activity. However, manipulating specific circuits within complex brain areas poses significant challenges due to heterogenous cell types with varying inherent sonosensitivity. Building on previous gas‐vesicle‐assisted ultrasound neuromodulation studies, we investigate an in vivo nanobubble‐enhanced sonogenetic strategy, termed mechanogenetics, for circuit‐level neural modulation. Application of this approach to distinct brain regions increased electromyography responses following motor‐cortex stimulation and enhanced neural activity in deep‐brain regions. Importantly, mechanogenetics enables us to modulate neural circuits controlling feeding behavior by specifically stimulating the mouse lateral hypothalamus (LH), with minimal disturbance in adjacent areas. The observed mechanogenetically‐induced decreases in feeding behavior were further found to be mediated by LH‐glutamatergic inputs to the ventral tegmental area. Furthermore, assessments of neural integrity, inflammation, and apoptosis support biosafety under the tested conditions. These findings demonstrate the feasibility of applying mechanogenetics to a behaviorally relevant deep neural circuit and provide a framework for localized, genetically targeted ultrasound neuromodulation.

## Graphical abstract

This study introduces mechanogenetics, a nanobubble‐enhanced sonogenetic strategy for selective modulation of feeding‐related neural circuits in the mouse lateral hypothalamus. Mechanogenetic stimulation modulates feeding behavior through glutamatergic projections from the lateral hypothalamus to the ventral tegmental area, highlighting its potential for circuit‐specific ultrasound neuromodulation and future therapeutic investigation of compulsive overeating.

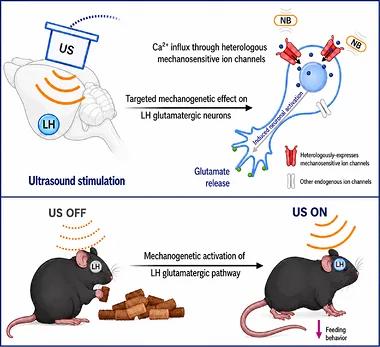

## 1 Introduction

Ultrasound (US) is a form of mechanical energy that can penetrate the intact skull and reach deep brain regions, enabling spatially and temporally controlled neuromodulation [1, 2, 3]. Low‐intensity ultrasound has shown promise in modulating the activity of the cortex and deep brain nuclei for treating various brain disorders in humans [4, 5, 6]. However, conventional transcranial ultrasound lacks the precision necessary to selectively modulate specific types of neurons [7]. Recently, sonogenetics has emerged as a technique that addresses this limitation by introducing exogenous mechanosensitive ion channels into selected cells [8, 9] to activate them specifically, but not nearby un‐transfected cells. Sonogenetic approaches have been evaluated in neurons [10, 11], brain tissue [12], *C. elegans* [8] and rodents [13, 14, 15, 16], and have been explored in disease‐relevant models, such as Parkinson's disease [17], diabetes [18], depression [19], and vision handicaps [16], highlighting its versatility and therapeutic potential. Leveraging the non‐invasive nature and exceptional tissue penetration depth of ultrasound, sonogenetics holds immense promise for translating remote‐control technologies into clinical applications.

The brain responds to mechanical cues through the cytoskeleton, extracellular matrix, cell adhesion proteins, and mechanosensitive ion channels [20, 21]. Several channels, including TRAAK [22], TRPA1 [11], TRPC6 [23], Piezo1 [24], and MscL [10] have been investigated as mediators of ultrasound neurostimulation. However, some of these channels are endogenously expressed with sonosensitivity not uniformly distributed across brain regions; the central amygdala (CeA), for example, demonstrated higher sonosensitivity through greater Piezo1 expression [24]. In addition, some channels exhibit limited intrinsic sensitivity, which may constrain the efficiency of ultrasound stimulation. These candidates suffer in contrast to optogenetics, which has gone through significant refinement and successfully identified enhanced light‐sensitive opsins (e.g., ChR2). Thus, the identification of suitably‐sonosensitive mechanosensitive ion channels is imperative for the future of ultrasound neuromodulation.

The complexity of the brain, which contains diverse cell types [25] with different intrinsic ultrasound sensitivities, presents an additional challenge for circuit‐specific sonogenetic modulation. Increasing mechanosensitive‐channel expression alone may not be sufficient to further sensitize target neurons and may raise biosafety concerns associated with higher viral loads. Additionally, given the anatomical challenges posed by large animals, characterized by thick skulls and extended paths to deep brain regions, there is an increased loss of ultrasound energy. Consequently, higher‐intensity ultrasound becomes imperative for effective brain stimulation in such subjects. Although increasing ultrasound exposure may improve neural activation, it could also expand the affected tissue volume and increase the likelihood of off‐target effects. Therefore, strategies that enhance local ultrasound responsiveness while retaining genetic cell‐type specificity are needed.

Acoustic enhancers that locally modify the acoustic microenvironment represent one potential approach to address these limitations [26, 27]. By lowering the activation threshold and enhancing sonosensitivity, external acoustic enhancers may achieve precise cellular targeting and accurate sonogenetics‐based neuromodulation, optimize the delivery and amplification of ultrasound energy while minimize off‐target effects. Previous studies have demonstrated the effectiveness of nanobubbles (NBs) in localizing and enhancing ultrasound effects [28, 29], which enables spatially‐precise neuromodulation. The introduction of NBs could reduce the ultrasound energy required for neural activation, thereby facilitating precise and targeted neuromodulation of neural circuits. In this study, we investigate whether combining MscL‐sonogenetics with nanotechnology (termed “mechanogenetics”) enables precise modulation of a behaviorally relevant, genetically defined deep‐brain circuit in vivo.

Here, we demonstrate the feasibility of applying NBs‐assisted sonogenetics to a behaviorally relevant neural circuit (Figure 1). Application of mechanogenetics to the lateral hypothalamus (LH) engaged glutamatergic neurons in awake, freely moving mice and altered feeding behavior. This behavioral effect was not observed in mice treated with NBs+ultrasound alone or with sonogenetics alone. Notably, the mechanogenetics‐induced reduction in feeding was reversed by blocking LH‐glutamatergic inputs to the ventral tegmental area (VTA). Under the tested conditions, no significant evidence of cell damage or inflammation was detected in the targeted brain regions. Together, these results support the feasibility of selective ultrasound neuromodulation to modulate feeding behavior in mice through the LH–VTA circuit.

**FIGURE 1:**
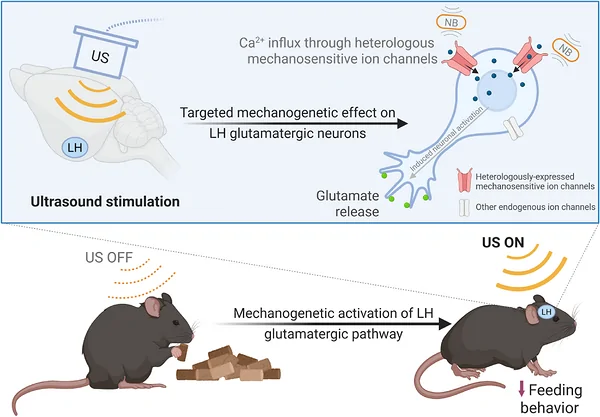
Schematic illustrating how ultrasound stimulation modulates mouse feeding behavior. Mechanogenetics selectively activates lateral hypothalamic (LH) glutamatergic neurons to regulate feeding.

## 2 Results

### 2.1 Nanobubble‐Enhanced Sonogenetic Neuromodulation

Given that the sensitization ability of nanobubbles (NBs) is critical, we first verified whether they could lower the acoustic pressure for sonogenetic neuromodulation. Our previous study demonstrated that NBs could enhance ultrasound neurostimulation by promoting the activation of mechanosensitive ion channels [24, 29]. We therefore hypothesized that introducing NBs as sono‐enhancers would reduce the acoustic energy required to elicit neural activation during sonogenetic stimulation (Figure 2A). Gas vesicles (GVs) are protein‐shell NBs known for their excellent stability, and we have previously shown that they are nontoxic to cultured cells and mice [30]. In this study, GVs were isolated from cultured *Anabaena* cyanobacteria and PEGylated to generate NBs for subsequent experiments. Transmission electron microscope (TEM) revealed that the NBs had an average width ranging from 50 to 100 nm and lengths from 200 to 600 nm (Figure 2B). To mitigate thermal accumulation, we employed pulsed ultrasound waves and observed only negligible changes in medium temperature following ultrasound exposure (Figure S1A–E).

**FIGURE 2:**
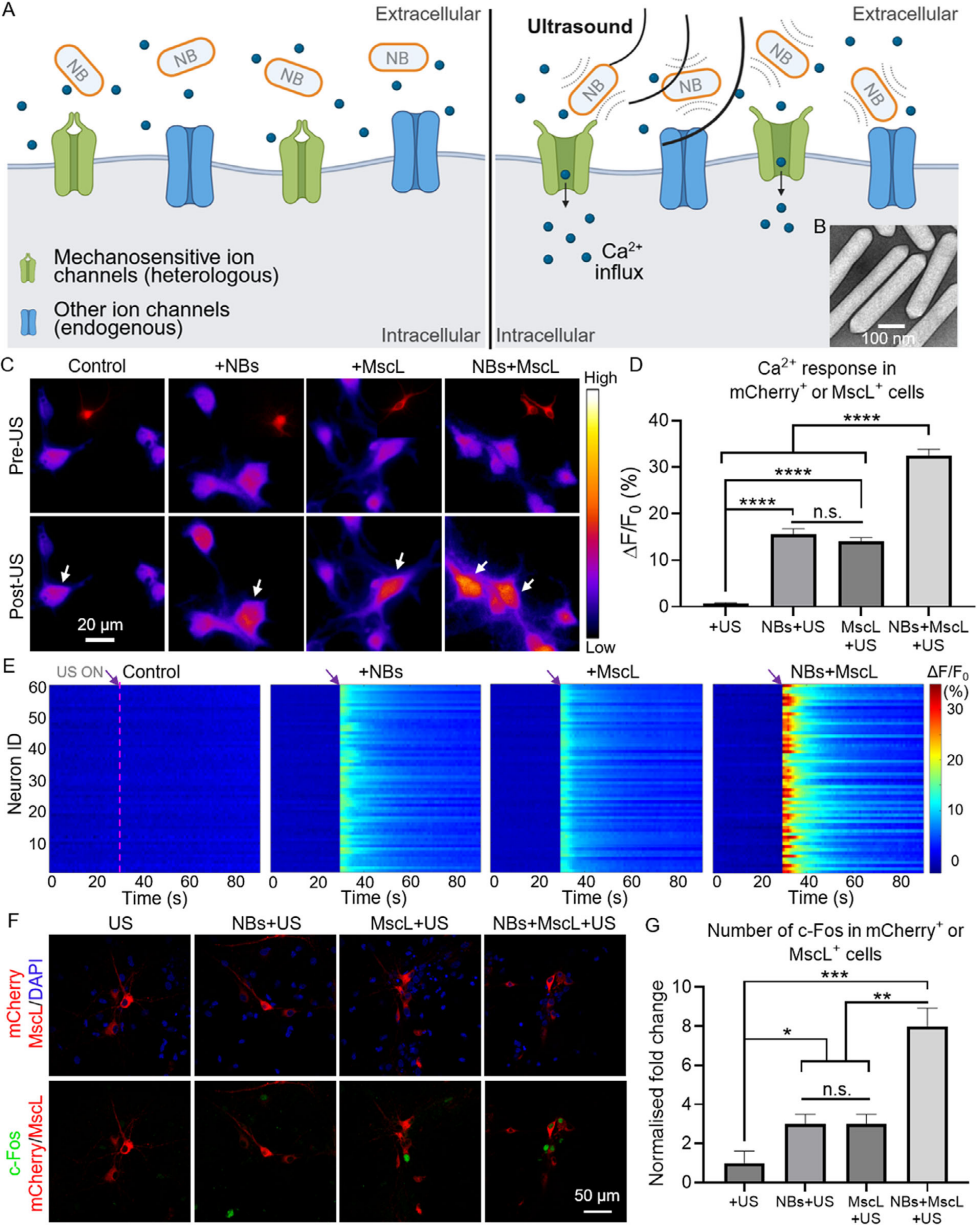
Nanobubbles decrease the acoustic pressure for sonogenetic neurostimulation. (A) Schematic illustration depicting the experimental scheme of mechanogenetics. A heterologous mechanosensitive channel (MscL) and nanobubbles were incorporated to enhance the ultrasound neurostimulation. (B) Transmission electron micrograph of nanobubbles. (C) Representative images showing calcium responses before and after ultrasound stimulation (Control and NBs conditions: neurons transduced with mCherry (control virus); red, mCherry‐ or MscL‐labeled neurons). (D) Quantification of calcium signals in mCherry^+^ or MscL^+^ neurons. (E) Temporal raster plots of fluorescence changes in mCherry‐ or MscL‐transduced neurons evoked by a single ultrasound pulse (purple arrow), with or without NBs. (F) Representative images of c‐Fos expression in mCherry‐ or MscL‐transduced cells following ultrasound stimulation, with or without NBs. (G) Quantification of nuclear c‐Fos staining in mCherry^+^ or MscL^+^ cells, with or without NBs, after ultrasound stimulation. Data are presented as mean ± SD from 5 independent experiments. n.s., not significant; *p < 0.05; **p < 0.01; ***p < 0.001; ****p < 0.0001 (one‐way ANOVA).

Additionally, we utilized low‐intensity ultrasound and aimed to enhance neuronal activation by introducing NBs. First, primary cultured neurons were transduced with MscL [9, 31], confirmed by fluorescence imaging and quantitative PCR analysis, in comparison to a transduction control (Figure 2C, Figure S1F). To test whether NBs sensitize MscL‐expressing neurons to ultrasound, we applied low‐intensity acoustic stimulation. Within the same field of view, MscL‐expressing cells (MscL^+^) showed much stronger calcium responses than non‐expressing cells, confirming functional MscL expression (Figure 2C). The maximal Ca^2+^ fluorescence increase (ΔF/F_0_) in the NBs+MscL+US condition reached 32.5 ± 3.1%, significantly higher than 14.0 ± 1.8% for MscL+US and 15.5 ± 2.7% for NBs+US group (Figure 2D). Moreover, NBs‐treated MscL^+^ neurons exhibited rapid and robust calcium responses to a single ultrasound pulse, whereas the other conditions showed markedly smaller or no responses, supporting a sensitizing effect of NBs (Figure 2E). Consistently, we observed significantly greater induction of nuclear c‐Fos (neural activation marker) [32, 33] in MscL^+^ neurons in the NBs+US condition compared with the MscL‐only, NBs‐only, and control group following ultrasound stimulation (Figure 2F,G). We also assessed cytotoxicity and NBs stability and observed no significant changes in cell viability or NBs concentration after ultrasound exposure (Figure S1G,H). Together, these results indicate that nanobubbles enhance the responsiveness of MscL‐expressing neurons to low‐intensity ultrasound stimulation, supporting the feasibility of targeted mechanogenetic neuromodulation.

### 2.2 Mechanogenetics Enabled Specific and Precise Neuromodulation In Vivo

To evaluate the in vivo feasibility of mechanogenetics, we first monitored electromyography (EMG) responses in the muscles during ultrasound stimulation of excitatory neurons of the mouse M1 motor cortex (Figure 3A). Among the ultrasound‐treated groups, NBs+MscL mice exhibited the highest EMG responses, with significantly greater contractions in NBs+MscL mice compared to the MscL‐alone group (Figure 3B,C), suggesting that NBs effectively enhance ultrasound brain stimulation. In the absence of ultrasound, baseline EMG activity in NBs+MscL mice was negligible (Figure S2A). Additionally, we assessed the effectiveness of NBs in enhancing neurostimulation by examining c‐Fos expression. Fluorescence imaging validated the presence of NBs (green) and MscL expression (red) in the specific cortical region (Figure S2B). Only the targeted brain area exhibited elevated c‐Fos levels following NBs/MscL+US stimulation, while the contralateral cortex showed no such changes, supporting the spatial precision of our stimulation strategy (Figure 3D). Importantly, the number of c‐Fos‐positive cells was significantly higher in the NBs+MscL group than in the MscL‐only or NBs‐only groups under identical ultrasound parameters (Figure 3D). As an additional control, mCherry^+^ mice receiving the same ultrasound stimulation showed c‐Fos levels at the injection site comparable to those in the contralateral cortex (Figure 3D). In particular, the number of c‐Fos‐positive MscL^+^ neurons in NBs+MscL mice was more than double (2.83‐fold) that of MscL‐alone mice following ultrasound stimulation (Figure 3E). We also quantified c‐Fos in MscL^−^ cells and found that the fraction of c‐Fos^+^ cells was higher in the MscL^+^ population than in the MscL^−^ population in both the MscL+US and NBs+MscL+US conditions, consistent with MscL expression increasing ultrasound sensitivity in the targeted population (Figure 3E). Together, these results indicate that mechanogenetics provides an effective, targeted means to activate excitatory neurons in the motor cortex, yielding reliable EMG responses.

**FIGURE 3:**
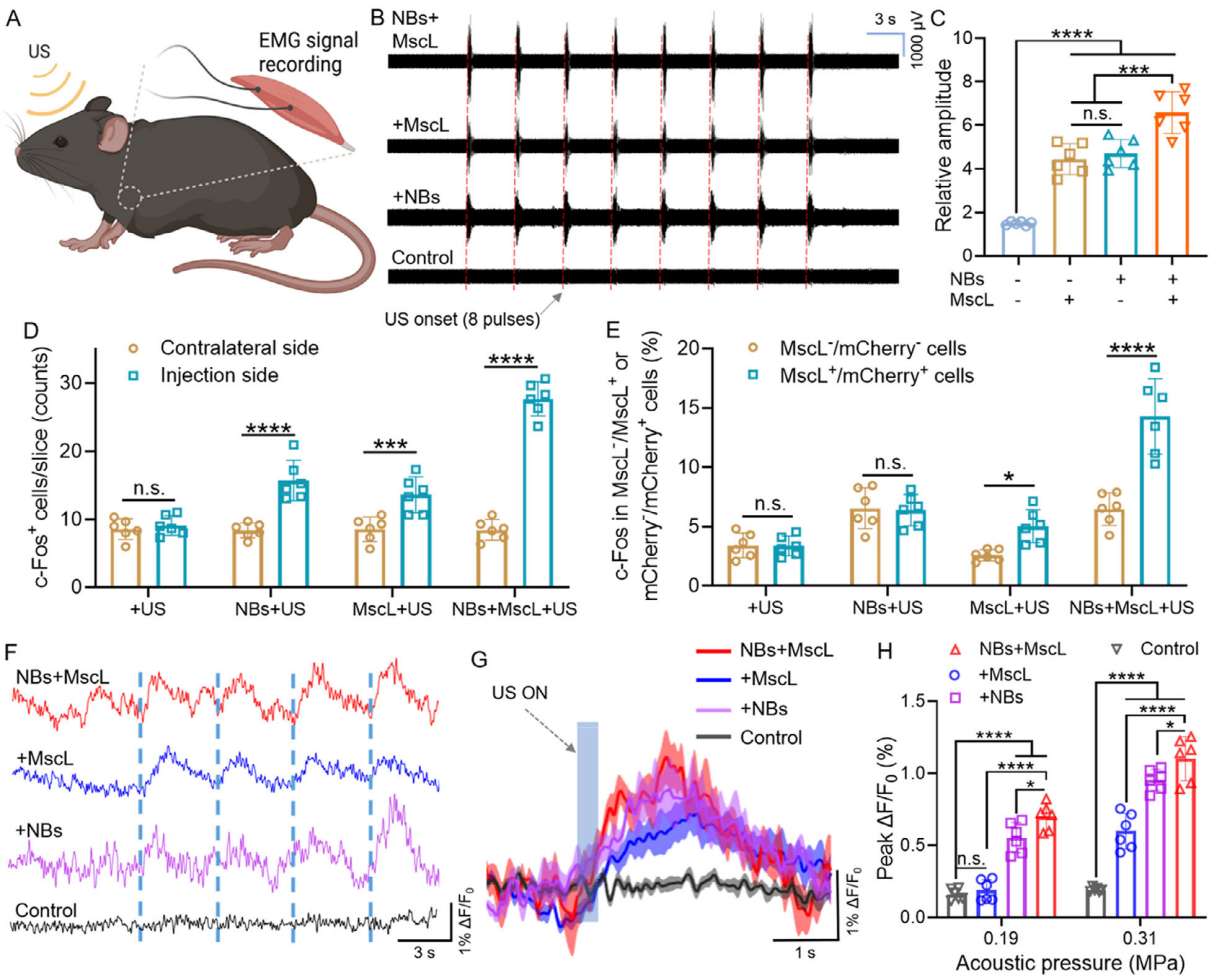
Mechanogenetics effectively triggered neural responses in vivo. (A) Schematic representation of electromyography (EMG) recordings. (B) Representative EMG signals showing muscular responses under low‐intensity ultrasound stimulation. (C) Relative amplitude of EMG responses following ultrasound stimulation. (D) Quantitative analysis of nuclear c‐Fos expression in mice across different treatment groups (0.19 MPa US). (E) Quantification and comparison of nuclear c‐Fos staining in MscL^+^ and MscL^−^ cortical cells under the indicated stimulation conditions. (F) Representative GCaMP6s fluorescence traces from the LH of mice under ultrasound stimulation. (G) Mean GCaMP6s fluorescence traces with the solid line representing the mean and the shaded region indicating SD. (H) Average peak of Ca^2+^ responses (∆F/F_0_) in response to ultrasound stimulation. Error bars indicate mean ± SD. Mice n  =  6 for each group. No significant differences (n.s.), *p < 0.05, ***p < 0.001, ****p < 0.0001, were analyzed using one‐way ANOVA for (C) or two‐way ANOVA for (D), (E), and (H).

To assess the efficiency of our mechanogenetics approach in deeper brain regions, we next monitored the neural activity in the mouse lateral hypothalamic (LH) area using fiber photometry (Figure S3A). LH excitatory neurons were transduced with CaMKIIα‐GCaMP6s, and an optical fiber was inserted to record neuronal calcium activity during ultrasound stimulation (Figure S3B). Remarkably, LH excitatory neurons in NBs+MscL mice exhibited repeated and reversible increases in GCaMP6s fluorescence, even under extremely low‐intensity ultrasound stimuli (Figure 3F–H). Importantly, in the control group of mice, we did not detect any calcium signals, suggesting that ultrasound alone is insufficient to activate neurons in LH (Figure 3F,G). There were no discernible repetitive patterns of fluorescence changes prior to the onset of ultrasound stimulation, ruling out the possibility of spontaneous responses. We further confirmed that injection of NBs+MscL had negligible effects on neuronal calcium activity (Figure S3C). In addition, the magnitude of responses in the NBs+MscL group demonstrated a general pattern of acoustic pressure dose dependence, showing significantly higher responses compared to MscL^+^ or NBs^+^ mice (Figure 3G,H). To assess response reliability independent of raw amplitude, we also computed z‐scored responses and observed a similar overall trend as with ΔF/F_0_ (Figure S3D,E). This finding highlights the enhanced sensitivity of our mechanogenetics approach relative to conventional MscL‐sonogenetics for ultrasound stimulation. The presence of NBs, coupled with the expression of mechanosensitive ion channels, provides a combined effect that enables more effective and precise activation of neurons in response to low‐intensity ultrasound stimulation.

### 2.3 Mechanogenetics Manipulates Feeding Behavior by Stimulating the LH Region

The lateral hypothalamus plays a crucial role in motivated behaviors [34, 35], including feeding, and may even contribute to the global obesity epidemic via the increasing prevalence of overeating disorders. Given the diverse cell types in the LH region, relying solely on sonogenetics may not suffice for modulating specific neural types due to the inherent sonosensitivity of these neurons. We aimed to leverage mechanogenetics to specifically modulate glutamatergic neurons in LH and control food intake in mice. To achieve this, we selectively induced the expression of MscL in LH excitatory neurons. As glutamatergic neurons are a type of excitatory neuron [36], we utilized either the CaMKIIα‐MscL‐mCherry or CaMKIIα‐mCherry virus to selectively target glutamatergic neurons in the LH (Figure 4A). The MscL/mCherry virus was injected into the LH, and after three weeks, NBs/saline were delivered to the same brain region. Following a few days of recovery, we conducted a feeding behavior test. A miniaturized ultrasound transducer was mounted on the mouse's head to allow free movement during the test in a behavioral box, and head motion in the food‐proximate region (pink circle) was identified as indicative of food exploration (Figure 4B,C). The mice were initially trained daily without ultrasound stimulation for 15 min for 4 days, followed by a 5‐min OFF, 5‐min ON, and 5‐min OFF cycle of ultrasound stimulation on day 5 (Figure S4A). We then examined the mice's food consumption and exploration time (Supplementary Movie S1).

**FIGURE 4:**
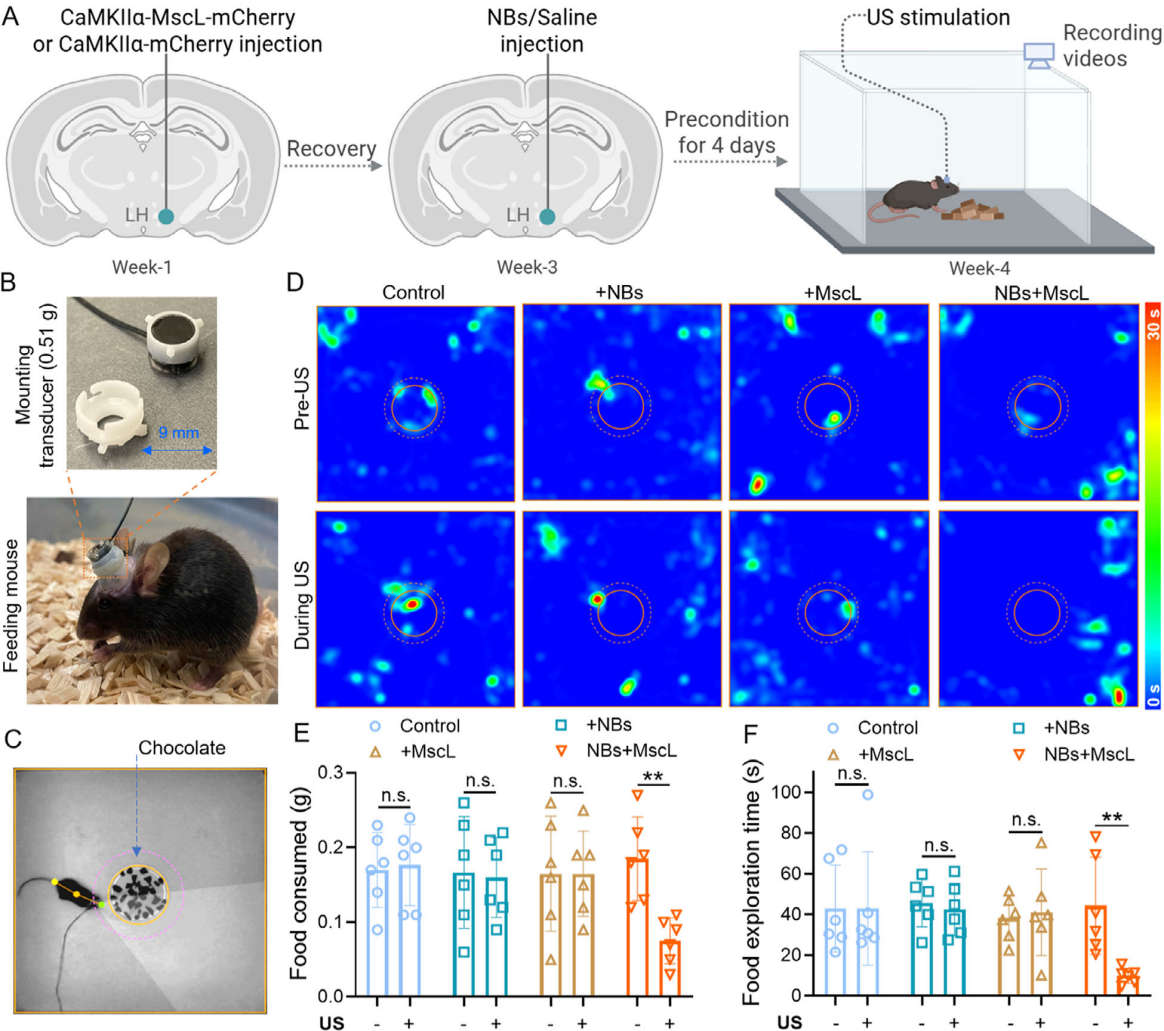
Modified feeding behavior in mice through mechanogenetics. (A) CaMKIIα‐MscL‐mCherry or CaMKIIα‐mCherry was injected into the LH area to express MscL‐mCherry or mCherry in excitatory neurons. After 3 weeks, nanobubbles or saline were injected into the same brain region. Following a brief training period, feeding behavior was tested. (B) Photograph depicting a mouse wearing a miniaturized ultrasound transducer, with the transducer adaptor having a diameter of 9 mm. (C) Image illustrating the analysis of mouse feeding behavior. (D) Representative occupancy heatmaps of mice before (top) and during US stimulation (bottom). (E) Food consumed by control mice, NBs‐injected mCherry^+^ mice, MscL^+^ mice, and NBs‐injected MscL^+^ mice before and during US stimulation. (F) Time spent in the food zone by control mice, NBs‐injected mCherry^+^ mice, MscL^+^ mice, and NBs‐injected MscL^+^ mice before and during ultrasound stimulation. N = 6 mice for each group, no significant differences (n.s.), **p < 0.01, analyzed using two‐way ANOVA for (E) and (F). Error bars in panels (E) and (F) represent the mean ± SD.

During the US OFF phase, no significant differences were observed in the food consumption and time spent in the food zone among the control, NBs, MscL, and NBs+MscL groups (Figure 4D–F). These findings indicate that neither virus nor NBs injection had an impact on basal feeding behavior. However, during the US ON stage, the NBs‐injected MscL^+^ mice showed reduced willingness to explore the food zone, while the control, NBs^+^, and MscL^+^ groups exhibited little change in their food zone behavior (Figure 4D). The NBs^+^ and MscL^+^ mice consumed a similar amount of food to the control mice during the US ON stage, whereas the NBs‐injected MscL^+^ mice consumed less food (Figure 4E). The time spent by the NBs+MscL mice exploring the food zone was also significantly decreased under US stimulation. In contrast, the control, NBs^+^, and MscL^+^ mice maintained consistent behavior in the food zone under US stimulation (Figure 4F), and there was a significant difference in the frequency of exploring the food zone during US stimulation between the NBs‐injected MscL^+^ group and the control, NBs^+^, and MscL^+^ groups (Figure 4D,F). We observed comparable feeding behavior in the control, NBs^+^, and MscL^+^ mice during US stimulation, with only the NBs‐injected MscL^+^ mice displaying reduced food consumption. Furthermore, our analysis revealed significant colocalization between the anti‐glutamate antibody (anti‐Glu, green) and mCherry signals, demonstrating high transfection efficiency of mCherry and MscL‐mCherry in the glutamatergic neurons of the LH region (Figure S4B). This provides further confirmation of our hypothesis that excitatory neurons were specifically targeted and activated by our strategy.

### 2.4 Mechanogenetics Effectively Activates Glutamatergic Neurons in the LH Area

To explore the effects of mechanogenetic stimulation and its spatial specificity, we monitored neural activity by assessing c‐Fos expression in relevant brain regions (Figure 5A). Increased c‐Fos signal was predominantly confined to the LH, supporting regional specificity (Figure 5B). To further evaluate activation of LH glutamatergic neurons, neurons were labeled with anti‐Glu [37]. After ultrasound stimulation, the NBs+MscL group exhibited a higher fraction of c‐Fos^+^ cells among anti‐Glu–positive neurons than the other groups, consistent with the behavioral outcomes. To assess off‐target activation, we further quantified c‐Fos induction in LH MscL^+^ neurons and compared it with MscL^−^ cells. c‐Fos colocalization was enriched in MscL^+^ neurons in the NBs+MscL+US (24.7% vs. 10.8%) and MscL+US (10.6% vs. 4.6%) group, whereas no significant difference was observed between mCherry^+^ and mCherry^−^ cells in the NBs+US (12.4% vs. 11.9%) or control group (3.2% vs. 4.8%) (Figure 5C). This supports the conclusion that MscL expression increases ultrasound response probability in the targeted population and strengthens the link between the behavioral effects and activation of the neuronal population of interest. Importantly, we did not observe a significant increase in c‐Fos expression in the auditory cortex of mice (Figure S5A–E), indicating that the observed activation was unlikely to be due to widespread auditory effects [38, 39]. Additionally, we did not find increased c‐Fos expression in other brain regions, such as the cortex and contralateral LH region (Figure S5A–D,F). These findings suggest that the ultrasound energy utilized in our study was sufficiently low, resulting in minimal impact on the other brain regions. The specificity of our approach in targeting the LH region without affecting the surrounding areas further underscores the precision and controlled nature of our ultrasound stimulation technique.

**FIGURE 5:**
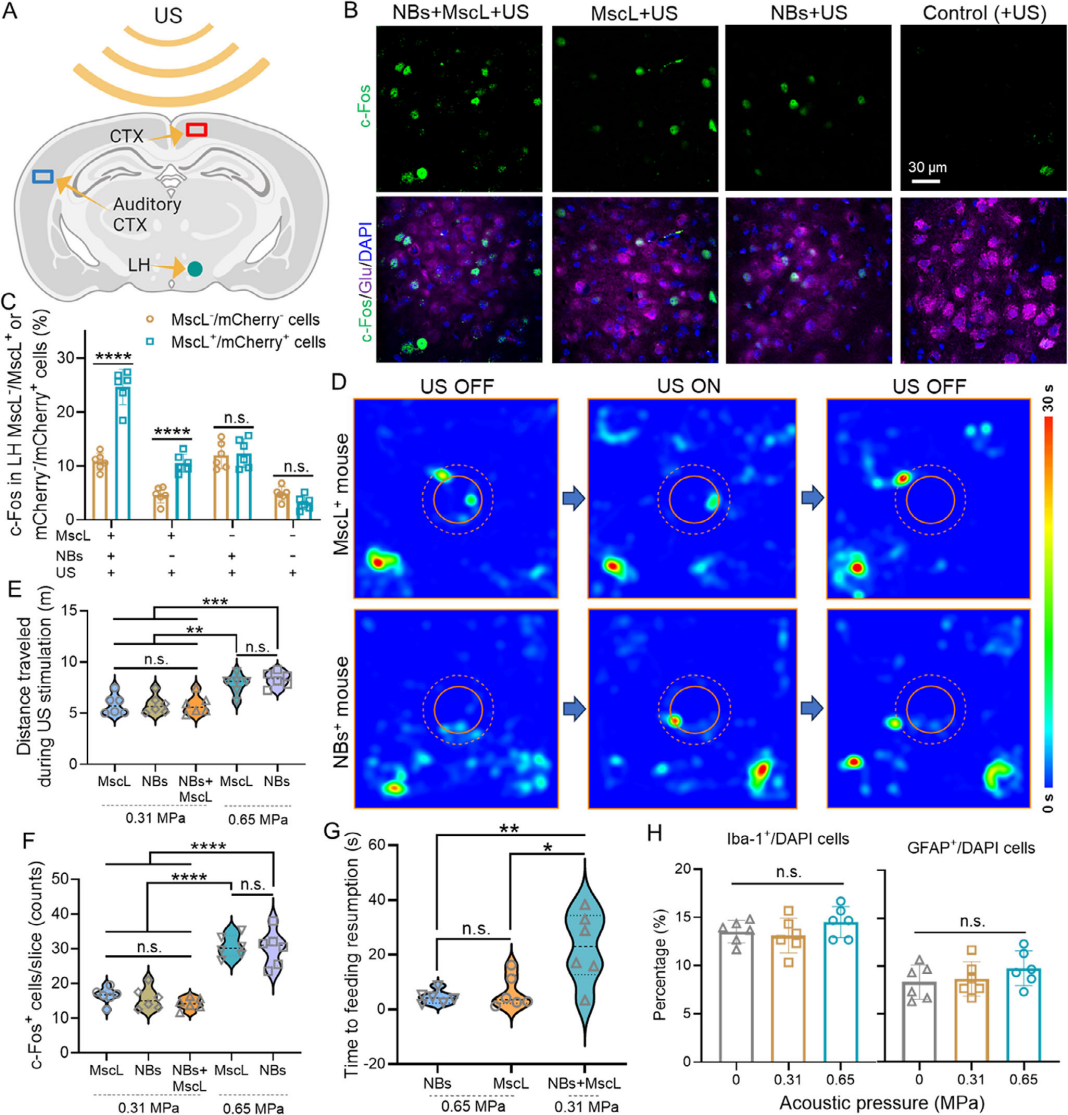
Selective activation of LH glutamatergic (Glu^+^) neurons by mechanogenetics. (A) Schematic illustration depicting the ultrasound‐treated brain areas, including the cortex (CTX), auditory cortex, and lateral hypothalamus (LH). (B) Representative immunofluorescence images displaying c‐Fos (green) / DAPI (blue) / anti‐Glu (purple) in the LH area under different treatment conditions. (C) Quantification and comparison of nuclear c‐Fos expression in MscL^+^/MscL^−^ or mCherry^+^/mCherry^−^ cells in the LH following the indicated stimulation conditions. (D) Representative heatmaps recorded from MscL^+^ and NBs^+^ mice pre‐US, during US, and post‐US with 0.65 MPa ultrasound application. (E) Measurement of the distance covered by the mice under different stimulated conditions. (F) Quantification of c‐Fos counts in the cortex of MscL^+^, NBs^+^ and NBs+MscL mice following treatment with various ultrasound energy levels. (G) The time taken by mice to resume feeding behavior after ultrasound cessation. (H) Quantification of cells per imaged slice, showing the percentages of Iba‐1– or GFAP‐positive cells relative to DAPI‐stained cells in the same field of view within the cortex across ultrasound energy levels. Error bars indicate mean ± SD for panels (C) and (H). Mice n  =  6 for each group, no significant differences (n.s.), *p < 0.05, **p < 0.01, ***p < 0.001, ****p < 0.0001, analyzed using two‐way ANOVA for (C) or one‐way ANOVA for (E‐H).

We further investigated whether increasing the US intensity could evoke similar feeding behavior in MscL^+^ or NBs^+^ mice. By increasing the applied ultrasound energy, we aimed to confirm whether the alterations in feeding behavior stemmed from the localized enhancement of ultrasound effects through our mechanogenetics approach. Elevating the acoustic pressure to 0.65 MPa produced a non‐significant trend toward changes in feeding‐related measures in MscL^+^ mice (Figure 4D, Figure 5D, Figure S6A–C, Supplementary Movie S2), but also broadly activated cortical neurons and altered locomotor activity in both MscL^+^ and NBs^+^ mice, leading to a less targeted behavioral response (Figure 5E,F, Figure S6D,E). Notably, in NBs+MscL mice at 0.31 MPa, feeding resumed after a longer latency (∼22.8 s) following ultrasound offset, whereas high‐intensity US produced much shorter suspensions (∼6.4 s in MscL^+^ mice and ∼4.5 s in NBs^+^ mice; Figure 5G). This effect may be linked to a high‐intensity ultrasound‐induced shock that halted feeding behavior in the mice. In addition, levels of the neuroinflammation markers Iba‐1 and GFAP tended to increase in both cortex and LH after high‐intensity stimulation compared with lower‐intensity ultrasound or control conditions, although these differences were not statistically significant (Figure 5H, Figure S7A–D, Figure 7F,G). Collectively, these results highlight the successful modulation of glutamatergic neuron activity within the LH region through our mechanogenetics system, leading to the regulation of feeding behavior in mice. Given the additional effects observed with higher‐intensity ultrasound, we opted to employ lower ultrasound energy for forthcoming investigations.

### 2.5 Mechanogenetics Effectively Triggers Glutamate Release in VTA and Disrupts Feeding Behavior

To further investigate the underlying mechanism of the behavioral changes triggered by mechanogenetic stimulation, we conducted fiber photometry to monitor the activity of neurons in the mouse ventral tegmental area (VTA). Our goal was to determine if this technique could selectively activate glutamatergic neurons and induce glutamate release. Initially, we introduced MscL/mCherry into the LH neurons, while the neurons in the ipsilateral VTA were transduced with the fluorescent glutamate sensor iGluSnFR3 (iGlu) [40]. Following a three‐week expression period, an optical fiber was implanted into the VTA to monitor alterations in glutamate activity via the sensor's fluorescence. (Figure 6A, Figure S8). We observed robust expression of iGlu (green) in the VTA and MscL (red) signals in LH neurons (Figure 6B). Upon stimulation of the LH with a pulse train of 0.31 MPa ultrasound, we observed rapid and synchronized increases in iGlu fluorescence in the VTA of NBs‐injected MscL^+^ mice, while there were minimal increases in MscL^+^ and NBs^+^ groups, and no detectable changes in the control group (Figure 6C–E). Furthermore, the glutamate signal responses triggered by mechanogenetics in response to ultrasound stimuli were consistent and demonstrated a swift response time of approximately 433 ms, slightly faster than both MscL‐sonogenetics (∼462 ms) and NBs+US‐treated (∼438 ms) groups (Figure 6C,F). Collectively, our data suggest that mechanogenetics can specifically induce glutamate secretion in neurons projecting to the VTA in vivo, leading to reduced feeding activity.

**FIGURE 6:**
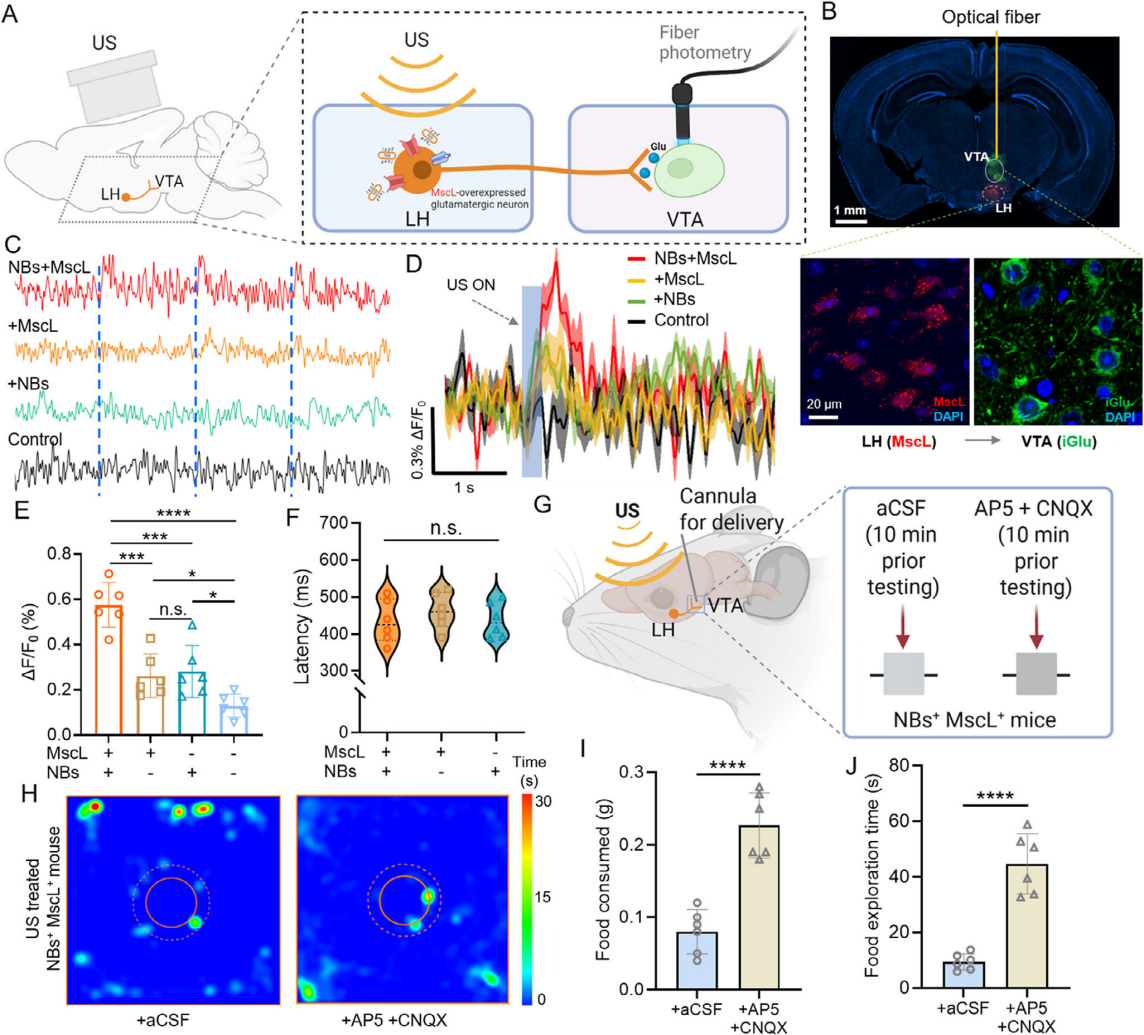
The release of glutamate in the VTA induced by mechanogenetics is responsible for the alterations in mice feeding behavior. (A) Schematic illustration depicting the NBs+US stimulation of the MscL^+^ mouse LH, while monitoring glutamate (Glu) release in the VTA via an optical fiber. (B) Representative fluorescence images showing the expression of the MscL (red) and glutamate sensor (iGlu, green) in the mouse LH and VTA, respectively. (C) Representative glutamate fluorescence responses in the mice VTA to ultrasound stimulation (blue dotted lines). (D) Averaged glutamate sensor signals in response to ultrasound stimulation. The light‐blue line indicates the onset of ultrasound. The solid line represents the mean, and the shaded region represents the SD. (E) Average peak of glutamate responses in different groups of mice. (F) Quantification of the latency between US onsets and the induced glutamate releases. (G) Schematic representation illustrating the injection of aCSF or AP5+CNQX into the mouse VTA. (H) Representative heatmaps recording the NBs+MscL mice under ultrasound stimulation with or without NMDA receptor antagonist (AP5) and AMPA receptor antagonist (CNQX). Quantification of food consumed (I) and measurement of the time spent exploring food (J) by the NBs+MscL mice during US stimulation. Error bars in panels (E), (I), and (J) represent the mean ± SD. Mice n  =  6 for each group, no significant differences (n.s.), *p < 0.05, ***p < 0.001, ****p < 0.0001, analyzed using one‐way ANOVA for (F) or two‐tailed unpaired t‐test for panels (E), (I), and (J).

We proceeded with a pharmacological approach to investigate the involvement of VTA glutamatergic receptors in the reduced feeding behavior observed following LH ultrasound stimulation in NBs+MscL mice. To achieve this, we conducted intra‐VTA injections of either artificial cerebrospinal fluid (aCSF) or a combination of NMDA receptor antagonist (AP5; 5 µg/µL) and AMPA receptor antagonist (CNQX; 5 µg/µL) prior to ultrasound stimulation [41] (Figure 6G). Our results revealed that ultrasound stimulation led to decreased feeding behavior in the presence of aCSF, but this feeding response could be blocked by the combination of NMDA and AMPA receptor antagonists (Figure 6H, Supplementary Movie S3). Moreover, we observed increased food consumption and feeding time in NBs+MscL mice treated with AP5+CNQX compared to mice administered with aCSF (Figure 6I,J). These findings indicate that our mechanogenetics approach effectively modulated mouse feeding behavior by selectively stimulating LH glutamatergic neurons to release glutamate in the VTA.

### 2.6 In Vivo Biosafety Assessment

Finally, we conducted investigations to assess the in vivo biosafety and biocompatibility of our system. The virus and NBs were injected into the specific brain region (LH area) by stereotaxic microinjection, following which the mice were treated with or without ultrasound stimulation. Throughout the post‐injection period, mice maintained stable body weights, and no deaths were observed, indicating negligible systemic toxicity (Figure 7A). As the NBs and virus were injected into the mouse brain, it was crucial to verify that they did not disrupt normal brain function. We found no significant changes in feeding behavior following the administration of NBs and the virus, suggesting that the system was safe and did not affect general activity such as feeding (Figure 4D–F).

**FIGURE 7:**
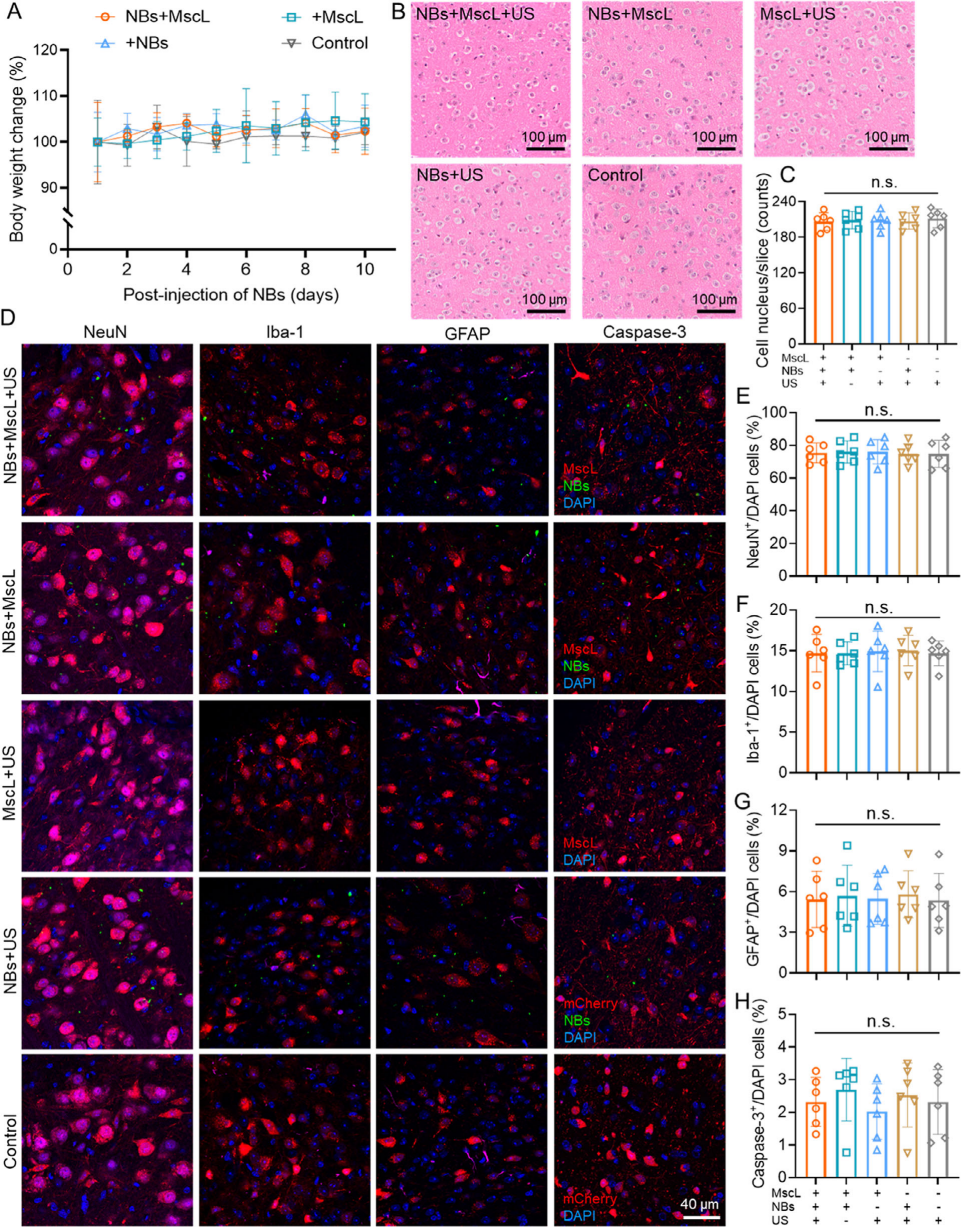
In vivo biosafety evaluation. (A) Measurement of mice's body weight during the 10‐day evaluation period (n  =  6 mice for all the groups). (B) Histological assessment of brain tissue sections in the LH area using H&E staining 24 h after ultrasound stimulation (n = 6). (C) Quantitative assessment of cell nuclei in the LH area of mice across various treatment groups. (D) Evaluation of neuronal integrity, inflammation, and apoptosis in the targeted brain region 24 h after ultrasound exposure, as determined by immunofluorescent staining for neurons (NeuN), microglia (Iba‐1), astrocytes (GFAP), and Caspase‐3. The injected NBs are shown in green, and DAPI‐stained nuclei are indicated in blue. Images represent 6 independent experiments (B) and (D). (E‐H) Cell counts in a 450 × 450 µm area per slice imaged, showing the percentage of cells positively stained for the indicated proteins NeuN (E), Iba‐1 (F), GFAP (G), and Caspase‐3 (H) relative to DAPI‐stained cells in the same field of view within the targeted brain region in mice (n  =  6). Error bars in panels (A), (C) and (E‐H) depict the mean ± SD. One‐way ANOVA for panels (C) and (E‐H) indicated no significant differences (n.s.).

In hematoxylin and eosin (H&E) histology assays, compared with the controls, minimal morphological differences and alterations in cell counts were observed in brain tissue (Figure 7B,C, Figure S9A) after the administration of mechanogenetics, MscL or NBs. Quantification of dark‐stained cells showed no significant differences among the treated groups, with consistently low percentages observed (Figure S9B), consistent with results from Caspase‐3 apoptosis staining. Additionally, there were no significant differences observed across samples in terms of neurons (NeuN; Figure 7D,E), microglia (Iba‐1; Figure 7D,F), astrocytes (GFAP; Figure 7D,G), or cell apoptosis (Caspase‐3; Figure 7D,H). The overexpression of MscL/mCherry through viral vector injection also did not result in any significant changes in the percentages of neurons, microglia, astrocytes, or apoptotic cells (Figure 7D–H). These findings indicate that, under the tested conditions and within the short‐term observation period, local NBs administration combined with MscL expression did not produce detectable local tissue toxicity. Additionally, ultrasound imaging was used to monitor the location and persistence of detectable NBs‐associated contrast signals in the mouse brain. Because collapsed NBs no longer generate detectable ultrasound contrast, the gradual decline in contrast signal over time (Figure S9C) may reflect NBs collapse, degradation or clearance. Notably, detectable echogenicity persisted for up to 11 days after injection, indicating that acoustically active NBs remained detectable in the brain over this period and providing a potential time window for ultrasound stimulation.

## 3 Discussion

Non‐invasive neuromodulation techniques with precise spatiotemporal resolution hold significant promise for advancing various realms of brain research. In this investigation, we have pioneered a groundbreaking approach for ultrasound‐based modulation of specific neural circuits utilizing nanobubble actuators, termed “mechanogenetics”. This technique overcomes the limitations of conventional ultrasound brain stimulation and sonogenetics by lowering the stimulation energy, allowing more precise neuromodulation. Crucially, mechanogenetics is orthogonal to other neuromodulation methods (electric, optical, magnetic) by leveraging the enhanced brain's sonosensitivity to activate mechanosensitive ion channels, facilitating controlled neurostimulation. Through the targeted activation of glutamatergic neurons, we successfully influenced feeding behavior in mice, resulting in reduced food intake.

Here, the NBs differ structurally from conventional spherical gas bubbles, exhibiting a cylindrical, gas‐filled morphology enclosed by a protein shell. In this study, we demonstrate that these cylindrical NBs can function as acoustic actuators to enhance ultrasound‐mediated neurostimulation. However, we do not exclude the possibility that spherical nanobubbles may also serve as acoustic enhancers. In comparison to the previous study of sonogenetics, where we activated the motor cortex of mice and triggered EMG signals using low ultrasound intensities, mechanogenetics demonstrated improved mechanical selectivity. Unlike traditional optical and magnetic stimulation methods, our strategy provides a straightforward approach to accurate mechanical neuromodulation. We note that the GCaMP6s signals in Figure 3F–H show small ΔF/F_0_ changes during ultrasound stimulation. Although the absolute amplitudes are modest, the responses are consistent and time‐locked to stimulation across trials and animals in the NBs+US, MscL+US, and NBs+MscL+US groups, rather than reflecting sporadic single‐trial fluctuations. Importantly, control animals show negligible ΔF/F_0_ changes, supporting that the observed signals are stimulus related. By targeting glutamatergic neurons in the lateral hypothalamus (LH), we observed a rapid suspension of feeding behavior, evident within one second of ultrasonic activation. This level of neuromodulation capability matches what has been demonstrated with optogenetic approaches [42] and surpasses the speed achieved by chemogenetics [43]. The slight delay in behavior change is not an intrinsic limitation of the technique but rather a result of the time required for entire neural circuits to respond and adapt.

To mitigate potential mechanical and thermal damage, we specifically selected low ultrasound intensities to minimize the risks associated with inertial cavitation and temperature increases. In our experiments, we intentionally restricted our ultrasound intensities to the lower range, with the maximum applied pressure being 0.65 MPa. Given that we utilized much lower ultrasound intensities and observed reversible neural activation, we anticipate that cell integrity is preserved following ultrasound stimulation. Additionally, we noted no significant changes in body weight among the mice post‐treatment, and immunofluorescent staining of brain slices revealed minimal inflammation and apoptosis following stimulation. These standard methods, including weight tracking and histological staining, did not indicate any signs of damage, further supporting the safety of our approach.

As ultrasound passes through larger animal brains, there is a greater loss of energy, necessitating the use of higher‐intensity ultrasound to achieve effective neuromodulation in deep brain regions. However, higher‐intensity ultrasound may lead to undesired effects on the animal's cortex. By employing localized ultrasound amplification, we can optimize the delivery of ultrasound energy to specific brain regions, minimizing potential off‐target effects and enhancing the translational potential of mechanogenetics‐based neuromodulation approaches. Unlike optogenetics, which may encounter challenges in displacing fibers due to the movement of large primate brains relative to the skull, ultrasound stimulation offers a promising alternative for deep brain stimulation. Additionally, the combination of nanobubbles and ultrasound can provide a “pre‐focused” ultrasound capability, allowing for targeted stimulation without the need for imaging guidance, thereby ensuring a consistent stimulation location. Locally distributed NBs may alter the acoustic microenvironment and enhance ultrasound‐induced mechanical perturbations, thereby facilitating neural activation within the targeted area. Furthermore, activating behaviors in non‐human primates often requires the coordinated activation of multiple distinct brain regions, each of which may exceed the volume covered by a single optical fiber. Ultrasound stimulation can easily target multiple brain areas, as the acoustic beam can encompass the necessary regions. The volume of stimulation can be precisely controlled by adjusting the amount and rate of nanobubble injection.

Although we successfully modulated feeding behavior, the current implementation requires minimally invasive intracranial delivery of both the viral vector and NBs. While stereotactic injection is widely used for localized delivery in animal brain studies, this requirement remains an important limitation and presents a barrier to clinical translation. The neuromodulation strategies based on piezoelectric and magnetoelectric nanomaterials [44, 45, 46] offer spatially localized stimulation without necessarily requiring genetic transduction; however, these approaches may provide limited intrinsic cell‐type specificity. Recent advances in focused‐ultrasound‐mediated blood–brain barrier (BBB) opening may provide a promising route for less invasive delivery of nanomaterials to the brain [47, 48]. These breakthrough in BBB opening has the potential to replace the invasive brain surgery currently utilized for injecting nanomaterials and viruses [49]. In addition, given the favorable acoustic responsiveness of NBs, they may serve as cargo carriers for ultrasound‐controlled multimodal neuromodulation, further expanding the versatility of the mechanogenetic platform. However, the feasibility, targeting precision, safety, and compatibility of these delivery strategies with the present mechanogenetic system require further investigation. Collectively, our findings establish the feasibility of this combined strategy for modulating the LH–VTA circuit and feeding‐related behavior and provide a framework for the future development of less invasive, cell‐type‐specific deep‐brain neuromodulation.

## 4 Methods

Our research complies with all relevant ethical regulations. The study protocol (23‐24/663‐BME‐R‐CRF) was approved by the Animal Subjects Ethics Sub‐Committee (ASESC) of The Hong Kong Polytechnic University.

### 4.1 Nanobubbles Preparation and Acoustic Field Characterization

*Anabaena flos‐aquae* was cultured in sterile BG‐11 medium at 25 °C under white lighting with a 14/10‐hour light/dark cycle [50, 51, 52, 53]. Gas vesicles (GVs) were extracted through hypertonic lysis by rapidly adding a sucrose solution to achieve a final concentration of 25%. After lysis, GVs were isolated by centrifugation at 400 x *g* for 3 h, purified by 3 rounds of the same procedure, followed by storage in PBS at 4 °C for further study. The concentration of GVs was determined by measuring the optical density at 500 nm (OD_500_) by a UV‐visible spectrophotometer [54, 55, 56]. To improve biocompatibility, we grafted the PEG on the GVs surface following our previous protocol [28], thereby generating nanobubbles (NBs) for utilization in this study. First, EDC/NHS was added to a PBS solution containing PEG‐amine and stirred at room temperature for 2 h. GVs were then added dropwise to the pre‐mixed solution, followed by stirring at 4 °C for an additional 24 h. PEG was conjugated to the GVs through EDC/NHS‐mediated amide‐bond formation. The resulting mixture was transferred to 2 mL tubes and washed four times with PBS to remove excess reagents. For fluorescence visualization, NBs were first biotinylated with Sulfo‐NHS‐Biotin for 20 min at room temperature. Excess biotinylation reagent was removed by three rounds of centrifugal flotation followed by PBS washing. The biotinylated NBs were then incubated with Alexa Fluor 488‐conjugated avidin (A21370, Invitrogen) for 30 min at room temperature. Unbound fluorescent avidin was removed by repeated centrifugal flotation and PBS washing before the labeled NBs were used in subsequent experiments.

For acoustic field characterization, a flat transducer with a center frequency of 1.0 MHz was utilized in this study. Ultrasonic pulses were generated using a function generator (AFG251, Tektronix) and amplified by a power amplifier (A075, Electronics & Innovation Ltd.). Each ultrasound stimulus consisted of 300 tone‐burst pulses at a center frequency of 1.0 MHz and a pulse‐repetition frequency of 1 kHz. Given the substantial differences in acoustic environments between cultured cells in vitro and brain tissue in vivo, including skull‐induced attenuation and scattering, the ultrasound parameters were adjusted accordingly for the in vivo experiments. Specifically, a duty cycle of 10% was used for in vitro experiments, whereas a duty cycle of 30% was used for in vivo experiments. Acoustic pressures were measured as peak negative pressures in degassed water using a hydrophone. Unless otherwise specified, low acoustic pressures ranging from 0.15 to 0.31 MPa were applied. The acoustic pressure distribution was characterized by point‐by‐point scanning using a computer‐controlled three‐axis translation stage. To monitor temperature changes during ultrasound exposure, a thermal camera (Hikvision p20 max) was used to measure the temperature of cell‐culture medium in a 35 mm dish positioned above the transducer head under the ultrasound conditions used for the in vitro and in vivo experiments (Figure S1C,D). Temperature changes were negligible during the 30‐min exposure period (Figure S1E), indicating minimal thermal contribution under the tested conditions.

### 4.2 Primary Cortical Neuron Culture

Cells were grown inside a standard humidified cell culture incubator at 37 °C with 5% CO_2_. Neurons from rat embryos at embryonic day 18 were obtained following our previous methods [9, 57]. Briefly, cortices were dissected and treated with 0.25% trypsin for 15 min at 37 °C, followed by gentle mixing. The trypsin digestion was halted by adding Neurobasal medium (Gibco) supplemented with 10% fetal bovine serum and 1% penicillin‐streptomycin. The resulting mixture was centrifuged for 5 min at 1,000 rpm, and the supernatant was discarded. The cells were then resuspended in the medium and gently triturated using a pipette. The cell suspension was filtered through a 40 µm filter to obtain individual cells. Subsequently, the cells were plated at a density of 1 × 10^5^ cells/cm^2^ on confocal dishes coated with 1.0 kPa polyacrylamide (PA) gel pre‐coated with poly‐L‐lysine [58, 59]. After 24 h, the medium was changed to Neurobasal medium supplemented with 2% B27, 0.25% L‐Glutamine, and 1% Penicillin‐Streptomycin (all from Gibco). Half the medium was replaced every 2 – 3 days to maintain cell viability and growth. On day 7, cultured neurons were transduced with virus and utilized in experiments between DIV 10 – 12 (3 – 4 days post‐infection).

### 4.3 In Vitro Ultrasound Stimulation

For this study, we utilized a customized system that allowed simultaneous ultrasound stimulation and calcium imaging. The ultrasound stimulation system was aligned with a calcium imaging system to monitor the calcium responses of stimulated neurons. Ultrasound was delivered through a waveguide filled with degassed water, which was connected to the ultrasound transducer assembly. Cells were cultured on PA‐gel within a confocal dish, and NBs were added to the medium and gently mixed just before stimulation. Prior to cellular stimulation, we assessed the acoustic pressure and field generated by the setup using a hydrophone and confirmed that it produced a relatively uniform ultrasound field in the central region. By delivering ultrasound in brief bursts, these settings were chosen to mitigate thermal effects. For experiments that did not involve real‐time imaging, cells were treated inside a standard cell culture incubator, following the procedures described in our previous study [9].

### 4.4 Neuronal Calcium Imaging In Vitro

The culture medium was substituted with a working solution of Fluo‐4 AM (Invitrogen) in calcium solution (pH 7.4), and the cells were incubated in the dark at 37 °C for 30 min [60]. Subsequently, the excess dye was removed by rinsing the cells with fresh calcium solution prior to ultrasound stimulation. Calcium imaging was conducted using a modified inverted epifluorescence microscope. The excitation light was generated by a dual‐color LED, filtered, and directed toward the sample to illuminate the calcium sensor. To minimize phototoxicity, the LEDs were triggered at a frequency of 1 Hz and synchronized with sCMOS time‐lapse imaging. Confocal dishes containing dye‐loaded cells were positioned above the objective, and NBs were introduced into the media immediately before ultrasound stimulation. A camera captured intracellular Fluo‐4 AM images at defined time intervals, with excitation wavelengths of 494 nm for Fluo‐4 AM. The cellSens software was employed to establish communication and coordinate the operational sequence between the microscope and monochromator.

### 4.5 MTT Assay

Cell viability after 30 min of ultrasound stimulation, in the presence or absence of NBs, was assessed using an MTT assay. Neurons were seeded in 96‐well plates and subjected to the designated NBs and ultrasound treatments. After treatment, cells were incubated with MTT solution (0.5 mg/mL) for 3–4 h at 37 °C [61, 62, 63]. The medium was then removed, and the resulting formazan crystals were solubilized in DMSO with gentle shaking for 15 min. Absorbance was measured at 570 nm using an LEDTect 96 microplate reader.

### 4.6 Animal Care

Male C57BL/6 mice were obtained from Jackson Laboratories and maintained under standard housing conditions, with free access to food and water. Animals from the aforementioned groups were randomly assigned to treatment groups. All animal experiments were conducted in accordance with the guidelines of the Animal Subjects Ethics Sub‐Committee of the Hong Kong Polytechnic University and complied with the regulations outlined by the Department of Health—Animals (Control of Experiments) of the Hong Kong S.A.R. government. The welfare and ethical treatment of the animals were prioritized throughout the study.

### 4.7 Stereotactic Injection of Virus and Nanobubbles

Male C57BL/6 mice at 8 weeks of age were anesthetized with Ketamine and Xylazine (100 mg/kg and 10 mg/kg respectively) administered via intraperitoneal (IP) injection. The anesthetized mice were positioned in a stereotaxic apparatus, and ointment was applied to protect their eyes. Skin incisions were made to expose the skull. It has been reported that MscL expression efficiently sensitizes cells to ultrasound stimulation [64]. A volume of 0.5 µL of rAAV9‐CaMKIIα‐MscL‐mCherry/rAAV‐CaMKIIα‐mCherry and rAAV9‐CaMKIIα‐GCaMP6s (1 × 10^12^ v.g., (BrainVTA (Wuhan) Co. Ltd)) were then injected into the LH, or 0.5 µL rAAV9‐hsyn‐iGluSnFR3 (1 × 10^12^ v.g., (BrainVTA (Wuhan) Co. Ltd)) was injected into the VTA at a rate of 50 nL/min, with a 10‐min interval. The pipette was slowly withdrawn after the injection. Following the completion of the procedure, the skin tissue was sutured and disinfected, and the mice were placed on a heating pad for recovery. Following surgery, mice were returned to their home cages and allowed to recover for four weeks to ensure sufficient transgene expression before experiments. Experimental groups are designated as follows: MscL+Saline = MscL, mCherry+NBs = NBs, and mCherry+Saline = Control, unless otherwise specified.

For targeted brain regions, a volume of 1.0 µL of NBs (4 nM) or saline alone was delivered by stereotactic injection at a rate of 100 nL/min, with a 10‐min interval. After the injection, the puncture site was disinfected, and sutured, and the mice were returned to their housing areas for recovery. The following coordinates were used for targeting specific brain regions: motor cortex—AP: 0.25 mm, ML: ‐1.50 mm, DV: ‐1.00 mm; ventral tegmental area (VTA)—AP: ‐2.90 mm, ML: ‐0.50 mm, DV: ‐4.50 mm; lateral hypothalamus (LH)—AP: ‐2.3 mm, ML: ‐0.7 mm, DV: ‐5.1 mm.

### 4.8 Biosafety Assessment In Vivo

NBs/Saline and MscL‐mCherry/mCherry virus were injected into the targeted brain region, specifically the LH area, and the mice were returned to their housing areas after the injection. To evaluate the overall safety, we conducted daily monitoring of the body weight of the mice that received virus/NBs. Changes in body weight served as an indicator of overall health. Additionally, we performed hematoxylin and eosin (H&E) histology assays to assess any morphological differences in brain tissues [44]. For this, the anesthetized mice were perfused with PBS and 4% paraformaldehyde (PFA), and their brains were subsequently removed and incubated overnight at 4 °C. Tissue sections were embedded in paraffin, sliced, and stained with H&E for histological analysis. To further assess the safety at the cellular level, we performed immunofluorescent staining on the treated brain slices using NeuN, Caspase‐3, Iba‐1, and GFAP antibodies to examine apoptosis, glial reaction, and inflammation.

### 4.9 EMG Recording in Anesthetized Mice

Mice were sedated using 2% isoflurane, and eye ointment was applied to protect their eyes. To monitor the bioelectric potential variations in the mouse's muscle tissue, two EMG electrodes were inserted into the contralateral biceps brachii muscle, with a distance of 3–5 mm between them. An EMG ground wire was attached to the mouse's tail. The concentration of isoflurane was reduced to 0.5% before the EMG recording commenced. Each mouse underwent three rounds of ultrasound stimulation in the motor cortex, with each round consisting of 7–10 ultrasonic stimuli spaced 5 s apart. There was a 50‐s interval between each stimulation round for each mouse. EMG signals were captured using a multi‐channel signal acquisition apparatus (Medusa, Bio‐Signal Technologies).

The recorded EMG data were analyzed using MATLAB software (The MathWorks, Inc.). The raw EMG data were filtered using a 50 Hz notch filter and a 10 to 150 Hz bandpass filter. The start of the recording and a 200 ms window were designated as the “calm time”, and the signals during the stimulated period were adjusted relative to these mean‐filtered signals. A sliding window of 200 ms was used to calculate the upper root‐mean‐square envelope, which helped smooth the data. The means of the corrected data during the calm period were considered the signal baseline. A threshold of 1.2 times the baseline was set, and any signal exceeding this level for at least 40 ms was identified as a “spike”. The relative amplitude (A_peak_/A_base_) was calculated by dividing the peak value of the recognized EMG event by the signal baseline.

### 4.10 Fiber Photometry Recording

Anesthetized mice were secured in a stereotaxic apparatus, and a small section of the skull (0.5 mm × 0.5 mm) was removed after removing the hair and skin. An optical fiber was implanted into either the LH or VTA, and it was fixed to the skull using dental cement, allowing it to be set for 20 min. Following the implantation procedure, mice were returned to their original housing and given a few days to recover. Once the mice had recovered, they were anesthetized with isoflurane. Ultrasound gel was applied to the shaved area of the head to enhance acoustic coupling. The mice were then stimulated with three trials of ultrasound stimulation, with each trial consisting of 5–10 ultrasound stimuli delivered at 5‐s intervals. A rest period of 50 s was provided between each trial. The fluorescence of the GCaMP6s/iGluSnFR3 sensor was captured using a fiber photometry system (Thinker Tech Nanjing BioScience Inc). The excitation wavelength used for fiber photometry was 470 nm, and data were collected at a rate of 100 Hz and analyzed using a custom MATLAB script.

### 4.11 In Vivo Ultrasound Stimulation and Mice Behavior Recording

The experiment commenced by administering a 1.0 µL injection of NBs into the LH region of mice. The control group received an equal volume of saline injection in the same brain region. A custom‐made wearable ultrasound transducer operating at 1.0 MHz generated an acoustic pressure of 0.31 MPa with a 30% duty cycle, unless otherwise specified. These parameters were used for all ultrasound stimulation experiments, except where specified otherwise. The transducer was enclosed in a 3D‐printed cap designed to fit a mounting plate attached to the mouse's head (Figure 4B). The mounting plate was positioned above the targeted brain area. Prior to the behavioral assessment, the plate was loaded with degassed ultrasound gel, and the capped ultrasound transducer was inserted into the mounting plate. Following a period of acclimatization to the testing environment, the mice's responses to ultrasound stimulation were systematically evaluated within an open field box for each trial.

A Logitech camera was utilized to track the mice's motion behavior before, during, and after ultrasound stimulation. For assessing feeding activity, animals were placed in a 40 cm × 40 cm open‐field box with a food tray containing palatable food (high‐sweetness chocolate) positioned at the center of the arena to quantify the amount of food consumed, and the time spent in the designated food zone was assessed. Mice were allowed to freely explore for 5 min, and ultrasound stimulation was applied for 5 min when the mouse's head was near the food. During this period, they were allowed to freely explore the arena. To test the on‐demand reversible suppression of feeding, well‐fed mice were monitored for 5 min pre‐stimulation, 5 min under ultrasound stimulation, and 5 min post‐stimulation (a total of 15 min). Their spatial locations, time spent at the food zone, velocity, and behavior were recorded at 15 fps, and video recordings were obtained for individual mice in semi‐dark conditions (∼20 lux). We defined the food‐proximate region as a circular zone extending 1.5 cm from the food edge, unless otherwise specified. This threshold was pre‐specified based on the tracking geometry in adult C57BL/6 mice—approximately one snout length from the tracked head point to the nose tip—and is therefore intended to capture nose‐directed investigation rather than incidental proximity. We also analyzed larger food‐proximate regions extending to 2.0 cm and 2.5 cm and found that the food exploration time shows a consistent trend across thresholds within the same treatment group (Figure S10). The data were analyzed using the software ANY‐maze (ANYmaze Video Tracking System 7.20). All the animals were allowed to acclimate to the behavior room for at least 1 h before the behavioral testing, and they were habituated at least 3 days prior to the arena environment, including equipment operation sounds without stimulation for 15 min.

### 4.12 Immunofluorescent Staining

The mice were subjected to 30 min of ultrasound treatment while under anesthesia. Following the completion of ultrasound treatment, the mice were perfused transcardially with PBS and 4% PFA. The brains were removed and fixed in 4% PFA overnight, and 40 µm‐thick coronal slices were obtained using a vibratome. Targeted brain slices were collected and then blocked for 90 min in a blocking medium containing 0.3% Triton X‐100, 10% normal goat serum, and 1% BSA. Subsequently, the slices were incubated at 4 °C for 16 – 18 h with the primary antibodies. After three 10‐min washes with PBS, the slices were incubated with secondary antibodies for 2 h at room temperature. Following three additional washes with PBS, the slices were mounted on glass slides using Prolong Diamond Antifade Mountant with DAPI. Primary antibodies used included NeuN (MA5‐33103, Invitrogen, diluted 1:500), Iba‐1 (#17198, Cell Signaling Technology, diluted 1:500), GFAP (#12389, Cell Signaling Technology, diluted 1:400), Caspase‐3 (#9661, Cell Signaling Technology, diluted 1:400), Anti‐Glutamate (G6642, Sigma–Aldrich, diluted 1:100) and c‐Fos (#2250, Cell Signaling Technology, diluted 1:500). Secondary antibodies, diluted 1:1,000, were goat anti‐mouse IgG (H+L) Alexa Fluor 633 (A‐21050, Invitrogen), goat anti‐rabbit IgG (H+L) Alexa Fluor Plus 488 (A‐32731, Invitrogen) or goat anti‐rabbit IgG (H+L) Alexa Fluor 633 (A‐21071, Invitrogen). The number of c‐Fos^+^ nuclei, NeuN^+^, Iba‐1^+^, GFAP^+^, and Caspase‐3^+^ cells was quantified using ImageJ. The count of c‐Fos^+^ cells per 200 × 200 µm area of the slice was determined, while the other four proteins were counted using slices of 450 × 450 µm. The counting of c‐Fos^+^, NeuN^+^, Iba‐1^+^, GFAP^+^, and Caspase‐3^+^ cells was performed by an experimenter who was unaware of the group assignments. All brain slices were imaged using a confocal microscope (TCS SP8, Leica) or a live‐cell fluorescence microscope (Nikon Eclipse Ti2‐E) at the ULS facilities at The Hong Kong Polytechnic University.

### 4.13 Statistical Analysis

GraphPad Prism was the software used for statistical analyses of all data and to prepare the graphs. Two‐tailed unpaired t‐tests, or one‐ or two‐way ANOVA were performed to determine statistical significance. P values below 0.05 were considered significant. Figure legends indicate the specific statistical test applied for each panel. Data are presented as mean ± S.D. as indicated.

## Author Contributions

X.H., and L.S. contributed to the conceptualization; X.H., and L.S. contributed to the methodology; X.H., J.J., and Z.S. contributed to the investigation; X.H., J.J., and Z.S. contributed to the data analysis; X.H., and L.S. contributed to the manuscript preparation; L.S. contributed to the funding acquisition.

## Conflicts of Interest

The authors declare no conflicts of interest.

## Supporting information




**Supporting File**:advs78177‐sup‐0001‐SuppMat.pdf.

**Supporting Video 1**: advs78177‐sup‐0002‐Movie1.mp4.

**Supporting Video 2**: advs78177‐sup‐0003‐Movie2.mp4.

**Supporting Video 3**: advs78177‐sup‐0004‐Movie3.mp4.

## Data Availability

The data that support the findings of this study are available on request from the corresponding author. The data are not publicly available due to privacy or ethical restrictions.

## References

[1] W. Legon , T. F. Sato , A. Opitz , et al., “Transcranial Focused Ultrasound Modulates the Activity of Primary Somatosensory Cortex in Humans,” Nature Neuroscience 17, no. 2 (2014): 322–329, 10.1038/nn.3620. 24413698

[2] Y. Tufail , A. Yoshihiro , S. Pati , M. M. Li , and W. J. Tyler , “Ultrasonic Neuromodulation by Brain Stimulation With Transcranial Ultrasound,” Nature Protocols 6, no. 9 (2011): 1453–1470, 10.1038/nprot.2011.371. 21886108

[3] W. J. Tyler , S. W. Lani , and G. M. Hwang , “Ultrasonic Modulation of Neural Circuit Activity,” Current Opinion in Neurobiology 50 (2018): 222–231, 10.1016/j.conb.2018.04.011. 29674264

[4] R. Beisteiner , E. Matt , C. Fan , et al., “Transcranial Pulse Stimulation With Ultrasound in Alzheimer's Disease—A New Navigated Focal Brain Therapy,” Advanced Science 7, no. 3 (2020): 1902583, 10.1002/advs.201902583. PMC7001626 32042569

[5] R. F. Dallapiazza , K. F. Timbie , S. Holmberg , et al., “Noninvasive Neuromodulation and Thalamic Mapping With Low‐Intensity Focused Ultrasound,” Journal of Neurosurgery 128, no. 3 (2017): 875–884, 10.3171/2016.11.JNS16976. PMC7032074 28430035

[6] H. Baek , K. J. Pahk , M.‐J. Kim , I. Youn , and H. Kim , “Modulation of Cerebellar Cortical Plasticity Using Low‐Intensity Focused Ultrasound for Poststroke Sensorimotor Function Recovery,” Neurorehabilitation and Neural Repair 32, no. 9 (2018): 777–787, 10.1177/1545968318790022. 30157709

[7] T. Liu , M. H. Choi , J. Zhu , et al., “Sonogenetics: Recent Advances and Future Directions,” Brain Stimulation 15, no. 5 (2022): 1308–1317, 10.1016/j.brs.2022.09.002. 36130679

[8] S. Ibsen , A. Tong , C. Schutt , S. Esener , and S. H. Chalasani , “Sonogenetics Is a Non‐Invasive Approach to Activating Neurons in Caenorhabditis elegans,” Nature Communications 6, no. 1 (2015): 8264, 10.1038/ncomms9264. PMC4571289 26372413

[9] Z. Qiu , S. Kala , J. Guo , et al., “Targeted Neurostimulation in Mouse Brains With Non‐Invasive Ultrasound,” Cell Reports 32, no. 7 (2020): 108033, 10.1016/j.celrep.2020.108033. 32814040

[10] J. Ye , S. Tang , L. Meng , et al., “Ultrasonic Control of Neural Activity Through Activation of the Mechanosensitive Channel MscL,” Nano Letters 18, no. 7 (2018): 4148–4155, 10.1021/acs.nanolett.8b00935. 29916253

[11] M. Duque , C. A. Lee‐Kubli , Y. Tufail , et al., “Sonogenetic Control of Mammalian Cells Using Exogenous Transient Receptor Potential A1 Channels,” Nature Communications 13, no. 1 (2022): 600, 10.1038/s41467-022-28205-y. PMC8828769 35140203

[12] T. Xu , D. Tan , Y. Wang , et al., “Targeted Sonogenetic Modulation of GABAergic Interneurons in the Hippocampal CA1 Region in Status Epilepticus,” Theranostics 14, no. 16 (2024): 6373–6391, 10.7150/thno.96598. PMC11488105 39431014

[13] Q. Xian , Z. Qiu , S. Murugappan , et al., “Modulation of Deep Neural Circuits With Sonogenetics,” Proceedings of the National Academy of Sciences 120, no. 22 (2023): 2220575120, 10.1073/pnas.2220575120. PMC10235981 37216521

[14] Z. Hu , Y. Yang , L. Yang , et al., “Airy‐Beam Holographic Sonogenetics for Advancing Neuromodulation Precision and Flexibility,” Proceedings of the National Academy of Sciences 121, no. 26 (2024): 2402200121, 10.1073/pnas.2402200121. PMC11214095 38885384

[15] Y.‐S. Huang , C.‐H. Fan , N. Hsu , et al., “Sonogenetic Modulation of Cellular Activities Using an Engineered Auditory‐Sensing Protein,” Nano Letters 20, no. 2 (2019): 1089–1100, 10.1021/acs.nanolett.9b04373. 31884787

[16] S. Cadoni , C. Demené , I. Alcala , et al., “Ectopic Expression of a Mechanosensitive Channel Confers Spatiotemporal Resolution to Ultrasound Stimulations of Neurons for Visual Restoration,” Nature Nanotechnology 18, no. 6 (2023): 667–676, 10.1038/s41565-023-01359-6. PMC10275757 37012508

[17] C.‐H. Fan , K.‐C. Wei , N.‐H. Chiu , et al., “Sonogenetic‐Based Neuromodulation for the Amelioration of Parkinson´s Disease,” Nano Letters 21, no. 14 (2021): 5967–5976, 10.1021/acs.nanolett.1c00886. 34264082

[18] H. Zhao , S. Xue , M.‐D. Hussherr , P. Buchmann , A. P. Teixeira , and M. Fussenegger , “Tuning of Cellular Insulin Release by Music for Real‐Time Diabetes Control,” The Lancet Diabetes & Endocrinology 11, no. 9 (2023): 637–640, 10.1016/S2213-8587(23)00153-5. 37620062

[19] T. Lei , Q. Xian , D. Li , et al., “Rapid Rescue Of Despair Behaviors By Sonogenetic Neuromodulation of the mPFC‐DRN Pathway,” molecular Psychiatry 31 (2026): 5279–5292. 10.1038/s41380-026-03616-1 PMC13442019 41998192

[20] W. J. Tyler , “The Mechanobiology of Brain Function,” Nature Reviews Neuroscience 13, no. 12 (2012): 867–878, 10.1038/nrn3383. 23165263

[21] D. G. Blackmore , D. Razansky , and J. Götz , “Ultrasound as a Versatile Tool for Short‐ and Long‐Term Improvement and Monitoring of Brain Function,” Neuron 111, no. 8 (2023): 1174–1190, 10.1016/j.neuron.2023.02.018. 36917978

[22] B. Sorum , R. A. Rietmeijer , K. Gopakumar , H. Adesnik , and S. G. Brohawn , “Ultrasound Activates Mechanosensitive TRAAK K + Channels Through the Lipid Membrane,” Proceedings of the National Academy of Sciences 118, no. 6 (2021): 2006980118, 10.1073/pnas.2006980118. PMC8017979 33542098

[23] Y. Matsushita , K. Yoshida , M. Yoshiya , et al., “TRPC6 is a Mechanosensitive Channel Essential for Ultrasound Neuromodulation in the Mammalian Brain,” Proceedings of the National Academy of Sciences (2024): 121, no. 50, e2404877121, 10.1073/pnas.2404877121. PMC11648612 39625977

[24] J. Zhu , Q. Xian , X. Hou , et al., “The Mechanosensitive Ion Channel Piezo1 Contributes to Ultrasound Neuromodulation,” Proceedings of the National Academy of Sciences 120, no. 18 (2023): 2300291120, 10.1073/pnas.2300291120. PMC10161134 37098060

[25] A. Joglekar , A. Prjibelski , A. Mahfouz , et al., “A Spatially Resolved Brain Region‐ and Cell Type‐Specific Isoform Atlas of the Postnatal Mouse Brain,” Nature Communications 12, no. 1 (2021): 463, 10.1038/s41467-020-20343-5. PMC7815907 33469025

[26] X. Hou , L. Liu , and L. Sun , “Precise Modulation of Cell Activity Using Sono‐Responsive Nano‐Transducers,” Biomaterials 314 (2024): 122857. 10.1016/j.biomaterials.2024.122857 39357155

[27] Y. Wu , X. Zhao , Y. Jiang , et al., “Microbubble‐Enhanced Ultrasound Stimulation of β‐Cells Improves Insulin Release and Glycemic Control in Mice,” Journal of Nanobiotechnology 24, no. 1 (2026): 77, 10.1186/s12951-025-03926-6. PMC12836977 41402805

[28] X. Hou , J. Jing , Y. Jiang , et al., “Nanobubble‐Actuated Ultrasound Neuromodulation for Selectively Shaping Behavior in Mice,” Nature Communications 15, no. 1 (2024): 2253, 10.1038/s41467-024-46461-y. PMC10937988 38480733

[29] X. Hou , Z. Qiu , Q. Xian , et al., “Precise Ultrasound Neuromodulation in a Deep Brain Region Using Nano Gas Vesicles as Actuators,” Advanced Science 8, no. 21 (2021): 2101934, 10.1002/advs.202101934. PMC8564444 34546652

[30] M. G. Shapiro , P. W. Goodwill , A. Neogy , et al., “Biogenic Gas Nanostructures as Ultrasonic Molecular Reporters,” Nature Nanotechnology 9, no. 4 (2014): 311–316, 10.1038/nnano.2014.32. PMC4023545 24633522

[31] Q. Xian , Z. Qiu , S. Kala , et al., “Protocol for the Sonogenetic Stimulation of Mouse Brain by Non‐Invasive Ultrasound,” STAR Protocols 2, no. 2 (2021): 100393, 10.1016/j.xpro.2021.100393. PMC7995654 33796870

[32] M. Sheng and M. E. Greenberg , “The Regulation and Function of c‐fos and Other Immediate Early Genes in the Nervous System,” Neuron 4, no. 4 (1990): 477–485, 10.1016/0896-6273(90)90106-P. 1969743

[33] J. Guo , Y. Wu , Z. Gong , et al., “Photonic Nanojet‐Mediated Optogenetics,” Advanced Science 9, no. 12 (2022): 2104140, 10.1002/advs.202104140. PMC9036029 35187865

[34] F. M. Reis , S. Maesta‐Pereira , M. Ollivier , et al., “Control of Feeding by a Bottom‐up Midbrain‐Subthalamic Pathway,” Nature Communications 15, no. 1 (2024): 2111, 10.1038/s41467-024-46430-5. PMC10920831 38454000

[35] E. H. Nieh , C. M. Vander Weele , G. A. Matthews , et al., “Inhibitory Input From the Lateral Hypothalamus to the Ventral Tegmental Area Disinhibits Dopamine Neurons and Promotes Behavioral Activation,” Neuron 90, no. 6 (2016): 1286–1298, 10.1016/j.neuron.2016.04.035. PMC4961212 27238864

[36] K. S. Matho , D. Huilgol , W. Galbavy , et al., “Genetic Dissection of the Glutamatergic Neuron System in Cerebral Cortex,” Nature 598, no. 7879 (2021): 182–187, 10.1038/s41586-021-03955-9. PMC8494647 34616069

[37] W. Zhou , Y. He , A. U. Rehman , et al., “Loss of Function of NCOR1 and NCOR2 Impairs Memory Through a Novel GABAergic Hypothalamus–CA3 Projection,” Nature Neuroscience 22, no. 2 (2019): 205–217, 10.1038/s41593-018-0311-1. PMC6361549 30664766

[38] T. Sato , M. G. Shapiro , and D. Y. Tsao , “Ultrasonic Neuromodulation Causes Widespread Cortical Activation via an Indirect Auditory Mechanism,” Neuron 98, no. 5 (2018): 1031–1041.e5, 10.1016/j.neuron.2018.05.009. PMC8127805 29804920

[39] H. Guo , M. Hamilton , S. J. Offutt , et al., “Ultrasound Produces Extensive Brain Activation via a Cochlear Pathway,” Neuron 98, no. 5 (2018): 1020–1030.e4, 10.1016/j.neuron.2018.04.036. 29804919

[40] A. Aggarwal , R. Liu , Y. Chen , et al., “Glutamate Indicators With Improved Activation Kinetics and Localization for Imaging Synaptic Transmission,” Nature Methods 20, no. 6 (2023): 925–934, 10.1038/s41592-023-01863-6. PMC10250197 37142767

[41] M. F. Barbano , S. Zhang , E. Chen , et al., “Lateral Hypothalamic Glutamatergic Inputs to VTA Glutamatergic Neurons Mediate Prioritization of Innate Defensive Behavior Over Feeding,” Nature Communications 15, no. 1 (2024): 403, 10.1038/s41467-023-44633-w. PMC10776608 38195566

[42] A. M. Stamatakis , M. Van Swieten , M. L. Basiri , G. A. Blair , P. Kantak , and G. D. Stuber , “Lateral Hypothalamic Area Glutamatergic Neurons and Their Projections to the Lateral Habenula Regulate Feeding and Reward,” The Journal of Neuroscience 36, no. 2 (2016): 302–311, 10.1523/JNEUROSCI.1202-15.2016. PMC4710762 26758824

[43] P. Kovács , T. Kitka , Z. K. Bali , et al., “Chemogenetic Inhibition of the Lateral Hypothalamus Effectively Reduces Food Intake in Rats in a Translational Proof‐of‐Concept Study,” Scientific Reports 14, no. 1 (2024): 11402, 10.1038/s41598-024-62014-1. PMC11102470 38762561

[44] T. Kim , H. J. Kim , W. Choi , et al., “Deep Brain Stimulation by Blood–brain‐Barrier‐Crossing Piezoelectric Nanoparticles Generating Current and Nitric Oxide Under Focused Ultrasound,” Nature Biomedical Engineering 7, no. 2 (2023): 149–163, 10.1038/s41551-022-00965-4. 36456857

[45] Y. J. Kim , N. Kent , E. Vargas Paniagua , et al., “Magnetoelectric Nanodiscs Enable Wireless Transgene‐Free Neuromodulation,” Nature Nanotechnology 20, no. 1 (2025): 121–131, 10.1038/s41565-024-01798-9. PMC11750723 39394431

[46] Q. Tang , D. Li , J. Chen , et al., “Ultrasound Active Piezoelectric Nanostimulators for Long‐Acting Wireless Deep Brain Electric Stimulation to Inhibit Epileptic Seizures,” Advanced Materials 38, no. 1 (2026): 20447, 10.1002/adma.202420447. 40923195

[47] K. Ogawa , N. Kato , M. Yoshida , et al., “Focused Ultrasound/Microbubbles‐Assisted BBB Opening Enhances LNP‐Mediated mRNA Delivery to Brain,” Journal of Controlled Release 348 (2022): 34–41, 10.1016/j.jconrel.2022.05.042. 35640764

[48] T. V. Parks , D. Szczupak , S.‐H. Choi , and D. J. Schaeffer , “Noninvasive Focal Transgene Delivery With Viral Neuronal Tracers in the Marmoset Monkey,” Cell Reports Methods 4, no. 2 (2024): 100709, 10.1016/j.crmeth.2024.100709. PMC10921014 38359822

[49] J. Blesa , J. A. Pineda‐Pardo , K. Inoue , et al., “BBB Opening With Focused Ultrasound in Nonhuman Primates and Parkinson's disease Patients: Targeted AAV Vector Delivery and PET Imaging,” Science Advances 9, no. 16 (2023): adf4888, 10.1126/sciadv.adf4888. PMC10115413 37075119

[50] L. Song , X. Hou , K. F. Wong , et al., “Gas‐Filled Protein Nanostructures as Cavitation Nuclei for Molecule‐Specific Sonodynamic Therapy,” Acta Biomaterialia 136 (2021): 533–545, 10.1016/j.actbio.2021.09.010. 34530143

[51] G. Wang , L. Song , X. Hou , et al., “Surface‐Modified GVs as Nanosized Contrast Agents for Molecular Ultrasound Imaging of Tumor,” Biomaterials 236 (2020): 119803, 10.1016/j.biomaterials.2020.119803. 32028170

[52] Y. Jiang , X. Hou , X. Zhao , J. Jing , and L. Sun , “Tracking Adoptive Natural Killer Cells via Ultrasound Imaging Assisted With Nanobubbles,” Acta Biomaterialia 169 (2023): 542–555, 10.1016/j.actbio.2023.07.058. 37536495

[53] X. Hou , Z. Qiu , S. Kala , et al., “Ultrasound Neuromodulation Through Nanobubble‐Actuated Sonogenetics,” BioRxiv (2020), 10.1101/2020.10.21.348870.

[54] A. Lakshmanan , G. J. Lu , A. Farhadi , et al., “Preparation of Biogenic Gas Vesicle Nanostructures for Use as Contrast Agents for Ultrasound and MRI,” Nature protocols 12, no. 10 (2017): 2050–2080, 10.1038/nprot.2017.081. PMC6185898 28880278

[55] Y. Yang , Z. Qiu , X. Hou , and L. Sun , “Ultrasonic Characteristics and Cellular Properties of Anabaena Gas Vesicles,” Ultrasound in Medicine & Biology 43, no. 12 (2017): 2862–2870, 10.1016/j.ultrasmedbio.2017.08.004. 28889941

[56] L. Song , G. Wang , X. Hou , et al., “Biogenic Nanobubbles for Effective Oxygen Delivery and Enhanced Photodynamic Therapy of Cancer,” Acta Biomaterialia 108 (2020): 313–325, 10.1016/j.actbio.2020.03.034. 32268236

[57] X. Hou , J. Jing , Z. Shi , Y. Jiang , and L. Sun , “Sono‐Mechanical Nanostructures‐Enabled Sustained Precise Ultrasound Brain Stimulation,” Nature Communications 17, no. 1 (2026), 3060, 10.1038/s41467-026-69710-8. PMC13039349 41730882

[58] L. A. Flanagan , Y.‐E. Ju , B. Marg , M. Osterfield , and P. A. Janmey , “Neurite Branching on Deformable Substrates,” Neuroreport 13, no. 18 (2002): 2411–2415, 10.1097/00001756-200212200-00007. PMC2408859 12499839

[59] A. K. Denisin and B. L. Pruitt , “Tuning the Range of Polyacrylamide Gel Stiffness for Mechanobiology Applications,” ACS Applied Materials & Interfaces 8, no. 34 (2016): 21893–21902, 10.1021/acsami.5b09344. 26816386

[60] X. Hou , Acoustic Nanoparticles Mediated Precise Brain Stimulation by Noninvasive Ultrasound, 2022.

[61] X. Hou , S. Cai , H. Cui , et al., “Tartrazine Clears Live Cells While Preserving Viability at High Refractive Indices and Osmolality,” Bioconjugate Chemistry 37, no. 7 (2026): 1329–1340, 10.1021/acs.bioconjchem.6c00164. PMC13377606 42275301

[62] X. Wang , L. Yang , H. Zhang , et al., “Fluorescent Magnetic PEI‐PLGA Nanoparticles Loaded With Paclitaxel for Concurrent Cell Imaging, Enhanced Apoptosis and Autophagy in human Brain Cancer,” Colloids and Surfaces B: Biointerfaces 172 (2018): 708–717, 10.1016/j.colsurfb.2018.09.033. 30245296

[63] X. Wang , L. Zhu , X. Hou , L. Wang , and S. Yin , “Polyethylenimine Mediated Magnetic Nanoparticles for Combined Intracellular Imaging, siRNA Delivery and Anti‐Tumor Therapy,” RSC Advances 5, no. 123 (2015): 101569–101581, 10.1039/C5RA18464A.

[64] Q. Xian , D. Li , X. Zhao , et al., “Impact of Endogenous Sonosensation on in vivo Sonogenetics,” Iscience 28, no. 12 (2025): 114030, 10.1016/j.isci.2025.114030. PMC12688701 41377656

