# Supplementary material for: Targeted Mechanogenetics to Modulate Specific Deep Neural Circuits
Source: Adv Sci (Weinh). 2026 Oct 6:e78177. Online ahead of print. doi: 10.1002/advs.78177 (PMC13643108; doi:10.1002/advs.78177)
Supplement: Supplementary file 1 [file ADVS-9999-e78177-s003.pdf]

Supporting Information for

Targeted mechanogenetics to modulate specific deep neural circuits

Xuandi Hou[1,2]*, Jianing Jing[1,2], Zhuohan Shi[1] and Lei Sun[1]*

[1]Department of Biomedical Engineering, The Hong Kong Polytechnic University, Hung Hom, Hong Kong SAR, China, 999077

[2]These authors contributed equally

*Correspondence to:
Xuandi Hou, Ph.D
Room MN106, Department of Biomedical Engineering,
The Hong Kong Polytechnic University,
Hong Kong SAR, People's Republic of China.
Phone: (852) 5510 1676, Email: xuandi.hou@connect.polyu.hk

Lei Sun, Ph.D
Room ST409, Department of Biomedical Engineering,
The Hong Kong Polytechnic University,
Hong Kong SAR, People's Republic of China.
Phone: (852) 2766 7663, Email: lei.sun@polyu.edu.hk

This PDF file includes:

Figures S1 to S10

Legends for Figures S1 to S10

Other supporting materials for this manuscript include the following:

Movies S1 to S3

Movie S1. The feeding behavior of NBs+MscL mouse during ultrasound stimulation. Movie playback is accelerated to five times the normal speed.

Movie S2. Higher intensity ultrasound induces decreased food intake in $MscL^+$ mouse. The playback speed of the video is accelerated to five times the normal speed.

Movie S3. Intra-VTA administration of the receptor antagonists AP5 and CNQX reduced avoidance responses induced by ultrasound stimulation in a NBs+MscL mouse. Movie playback is accelerated to five times the normal speed.

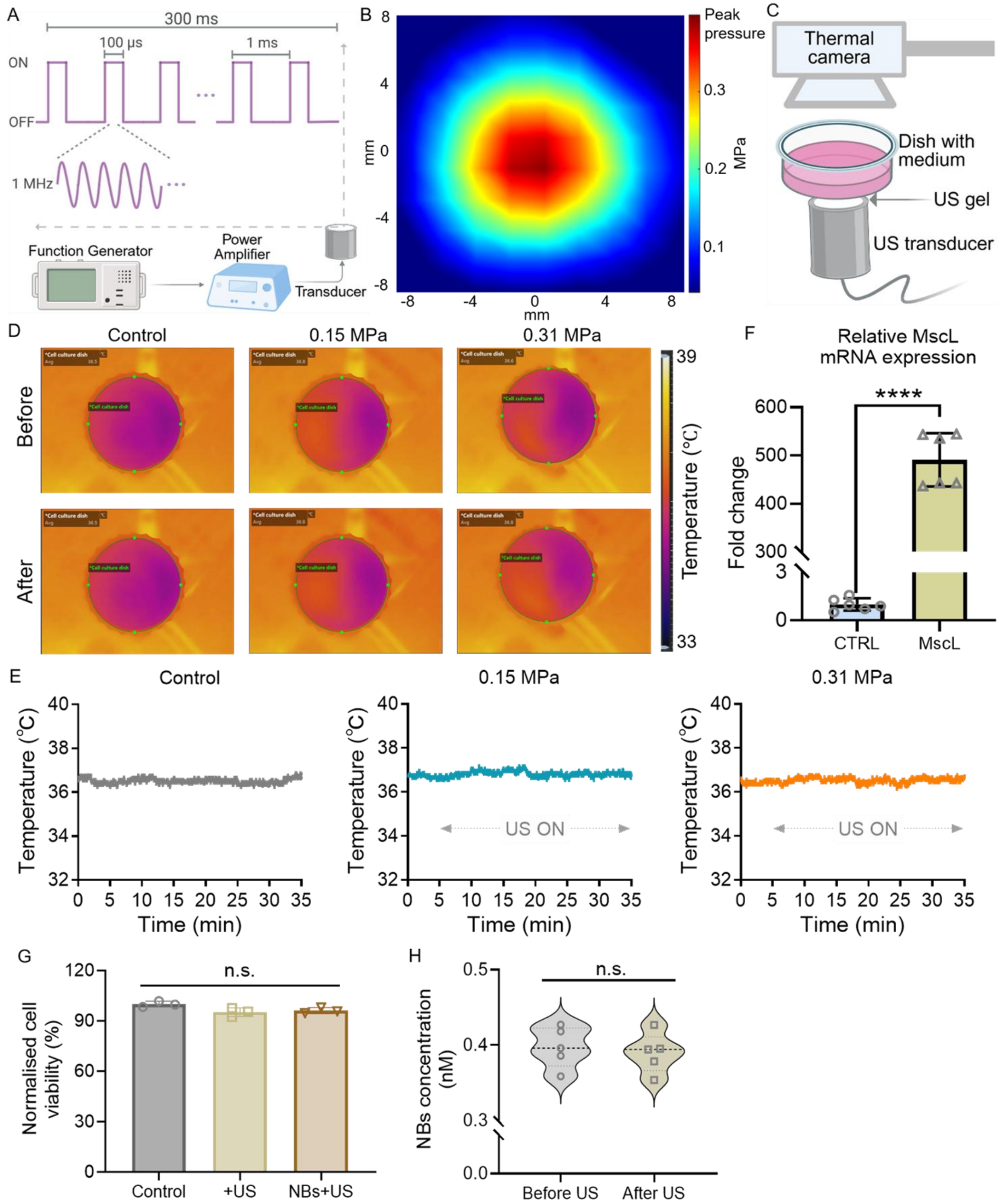

***Figure S1. Characterization of the acoustic field, viral transduction, cell viability and NBs stability**. (**A**) Schematic illustration of the ultrasound waveform used for stimulation. (**B**) The colormap illustrates the spatial profile of the acoustic pressure. (**C**) Schematic of the temperature-measurement setup during ultrasound*

*stimulation. (**D**) Thermal-camera images showing the ultrasound-induced temperature distribution in the cell-culture medium. (**E**) Mean temperature of the treated medium as a function of time. Ultrasound was applied at 1 MHz and 0.15 or 0.31 MPa with a 30% duty cycle, using 5-s intervals for 30 min at 37 °C. (**F**) Quantitative PCR analysis showing the expression of MscL in transfected cells compared to a mock-transfection control (CTRL) (normalized to GAPDH). (**G**) Cell viability following the indicated treatments was assessed using an MTT assay (n = 3). (**H**) Changes in NBs concentration before and after ultrasound exposure (n = 5). Data are presented as mean ± SD. Statistical analysis was performed using one-way ANOVA for (**G**) and two-tailed unpaired t-test for (**F**) and (**H**), n.s., not significant; ****p < 0.0001.*

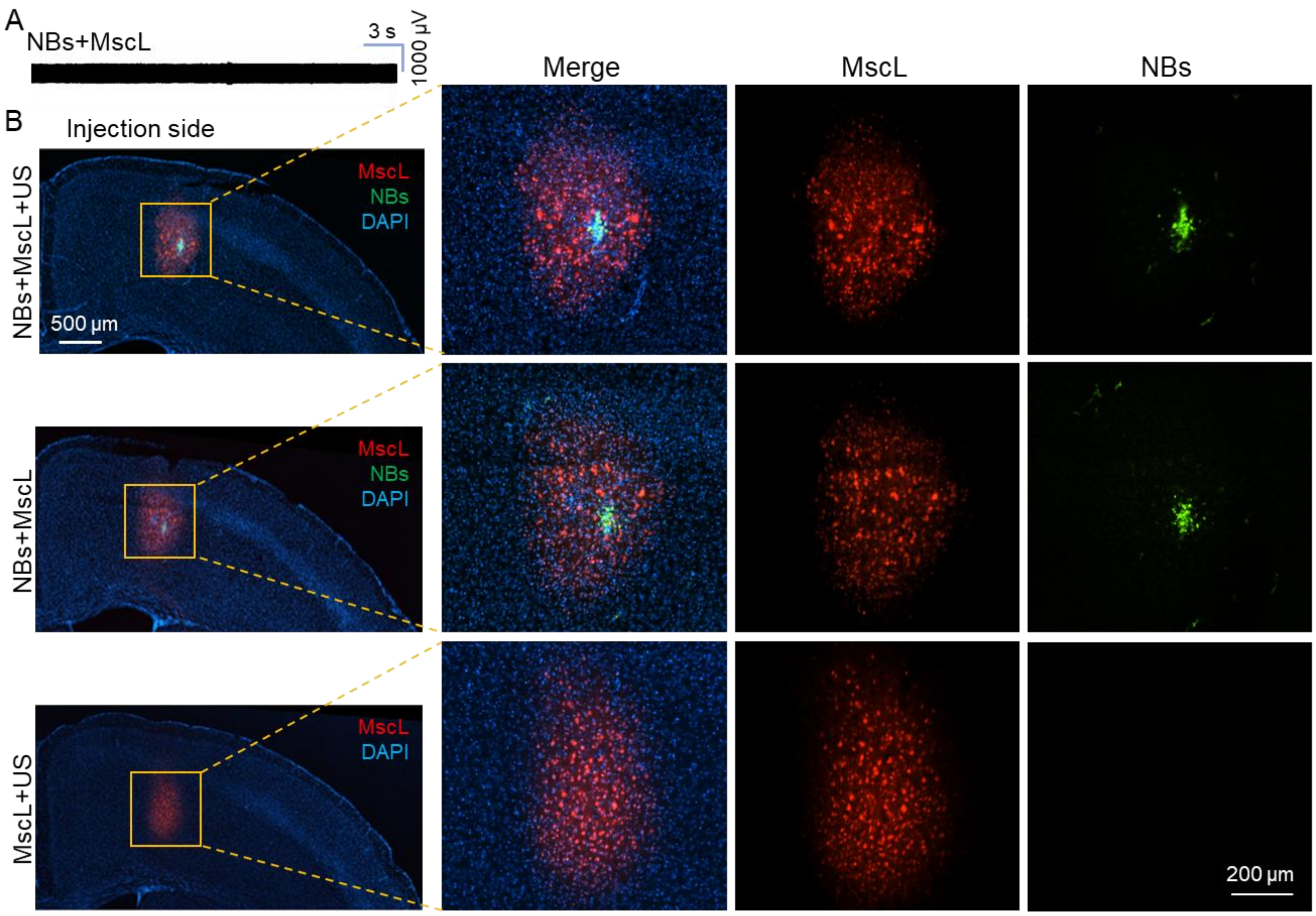

Figure S2. EMG recordings and fluorescence images of mouse brain sections. *(**A**) Minimal EMG activity in NBs+MscL mouse in the absence of ultrasound stimulation. (**B**) Low- and high-magnification images of mouse brain sections showing MscL expression (red) and NBs localization (green). Yellow squares mark the cortical injection site.*

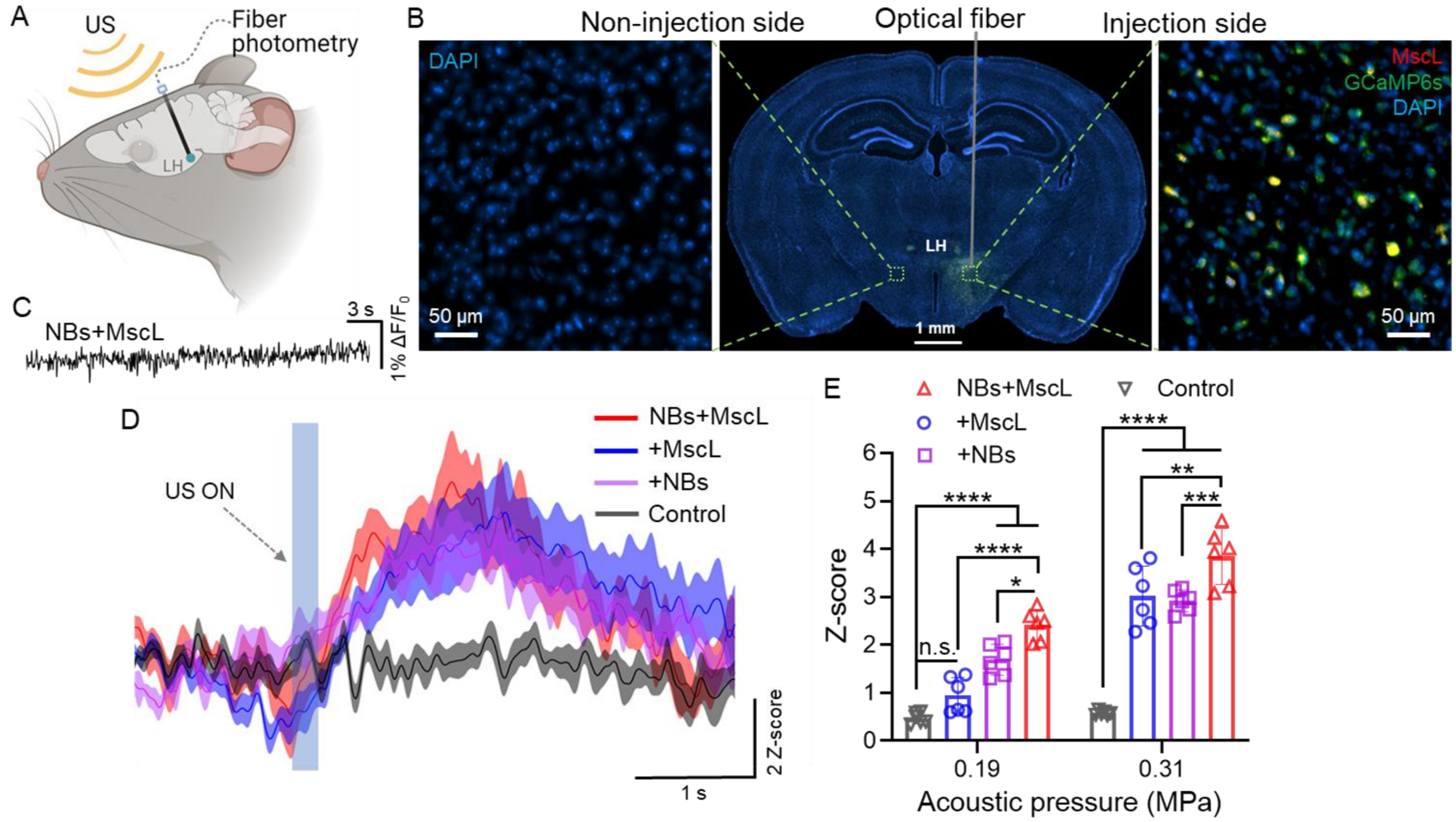

Figure S3. Fiber photometry recorded neural calcium activity in the LH region. *(**A**) Schematic illustrating the fiber photometry setup employed to measure LH neural Ca^{2+} dynamics during US stimulation. (**B**) Representative images displaying GCaMP6s expression in LH. (**C**) Minimal changes in GCaMP6s fluorescence in NBs+MscL mouse in the absence of ultrasound stimulation. (**D**) Mean GCaMP6s fluorescence traces (z-scored) under the indicated treatment conditions. (**E**) Average peak of Ca^{2+} responses (z-scored) evoked by ultrasound stimulation. Mice n = 6 for each group. Bars indicate mean ± SD. No significant differences (n.s.); *p < 0.05; **p < 0.01; ***p < 0.001; ****p < 0.0001 (two-way ANOVA).*

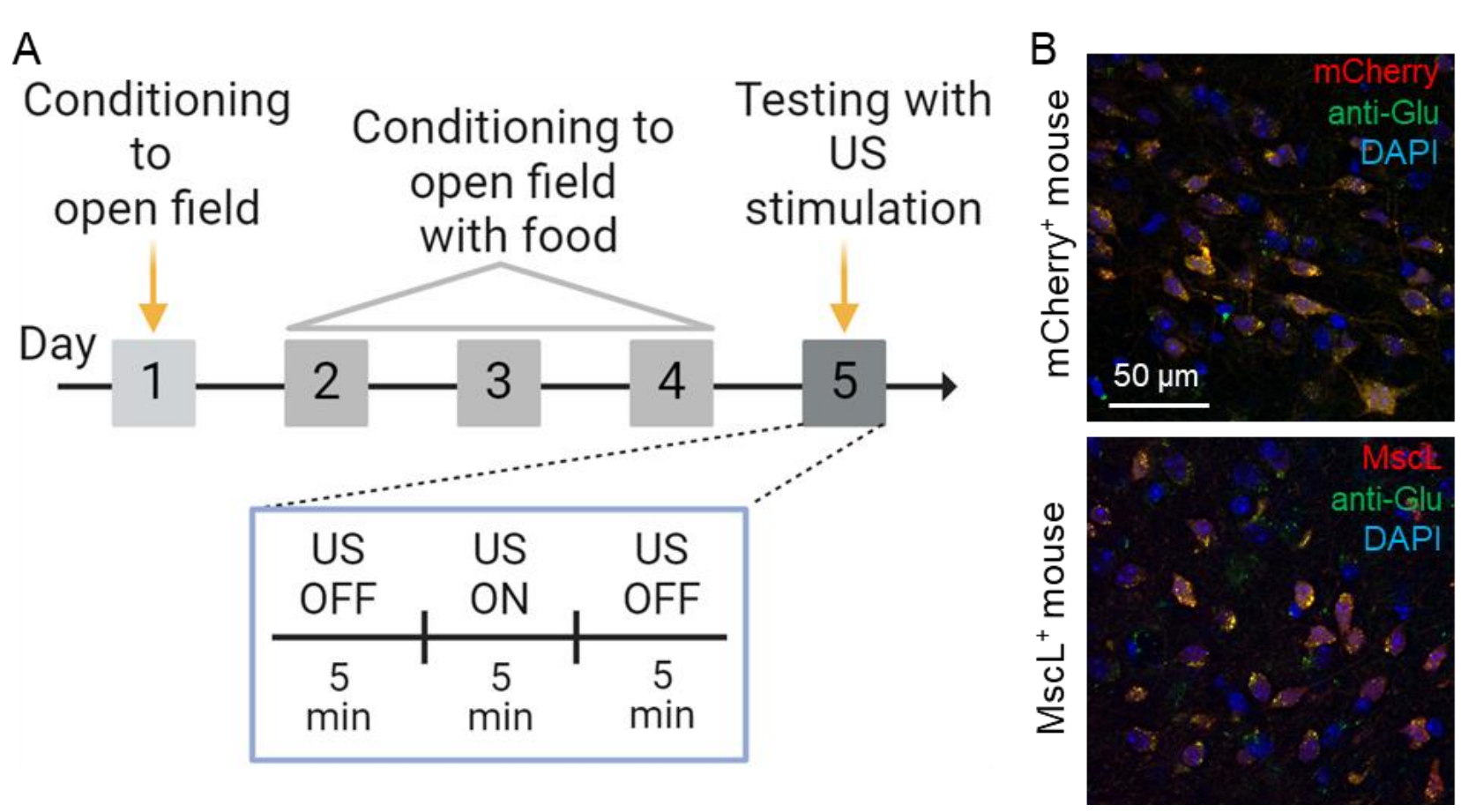

Figure S4. Evaluation of mice feeding behavior and verification of virus transduction. *(**A**) Timeline outlining the progression of events for the mice feeding experiments. (**B**) Immunofluorescence images demonstrate the co-localized expression of MscL/mCherry (red) and glutamatergic neurons (green) in the LH region of mice.*

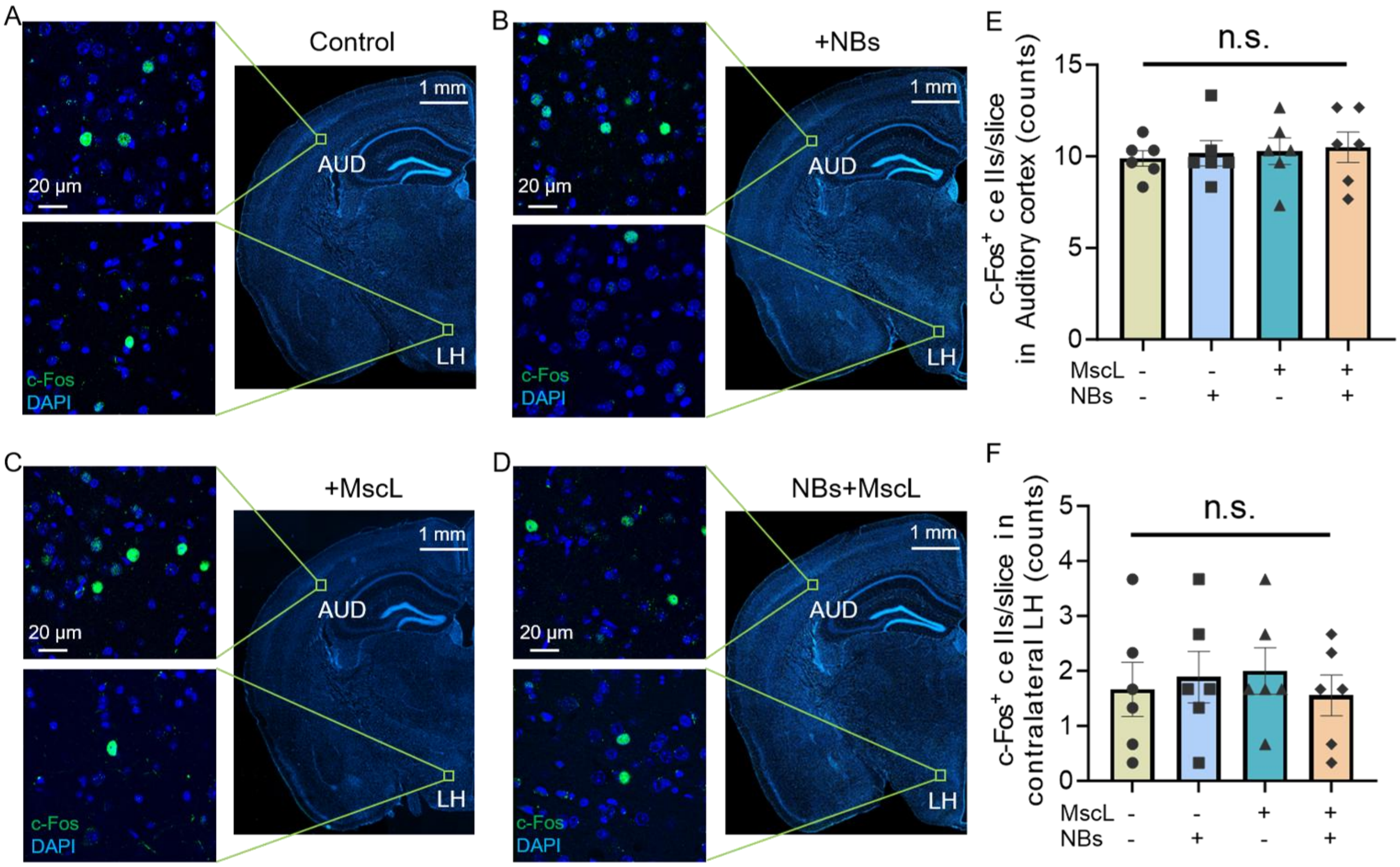

Figure S5. Assessment of ultrasound effects on other brain regions. *(**A**-**D**) Representative images displaying c-Fos staining in the auditory cortex (AUD) and contralateral LH region of mice following ultrasound brain stimulation. The sample size (n) for all groups was 6. (**E**-**F**) Quantitative analysis of c-Fos counts in brain slices. Error bars indicate mean ± SD. No significant differences (n.s.) were analyzed using one-way ANOVA.*

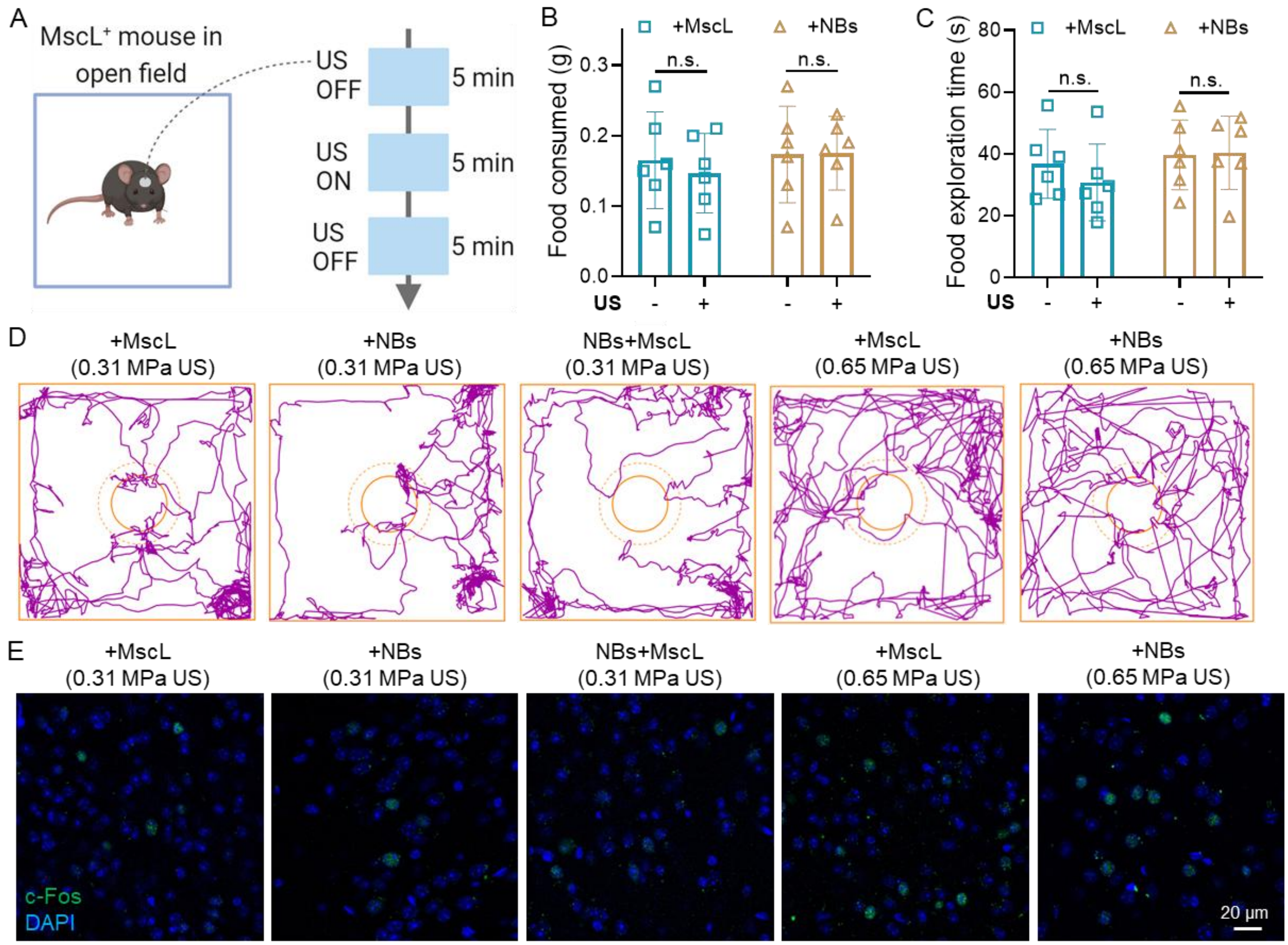

Figure S6. Assessment of the effects of different ultrasound stimulation on mouse behavior and c-Fos expression. *(**A**) Illustration depicting the experimental procedure. Quantification and comparison of food consumption (**B**) and food exploration time (**C**) in MscL⁺ and NBs⁺ mice under 0.65 MPa ultrasound stimulation. n = 6 mice per group. Statistical significance was assessed by two-way ANOVA for panels (**B**) and (**C**): n.s., not significant. Error bars indicate mean ± SD. (**D**) Track plots for mice under different ultrasound treatments (each trace lasting 5 minutes). (**E**) Representative images showing c-Fos expression in mice brains exposed to different ultrasound treatments. Images are representative of 6 independent biological replicates per condition.*

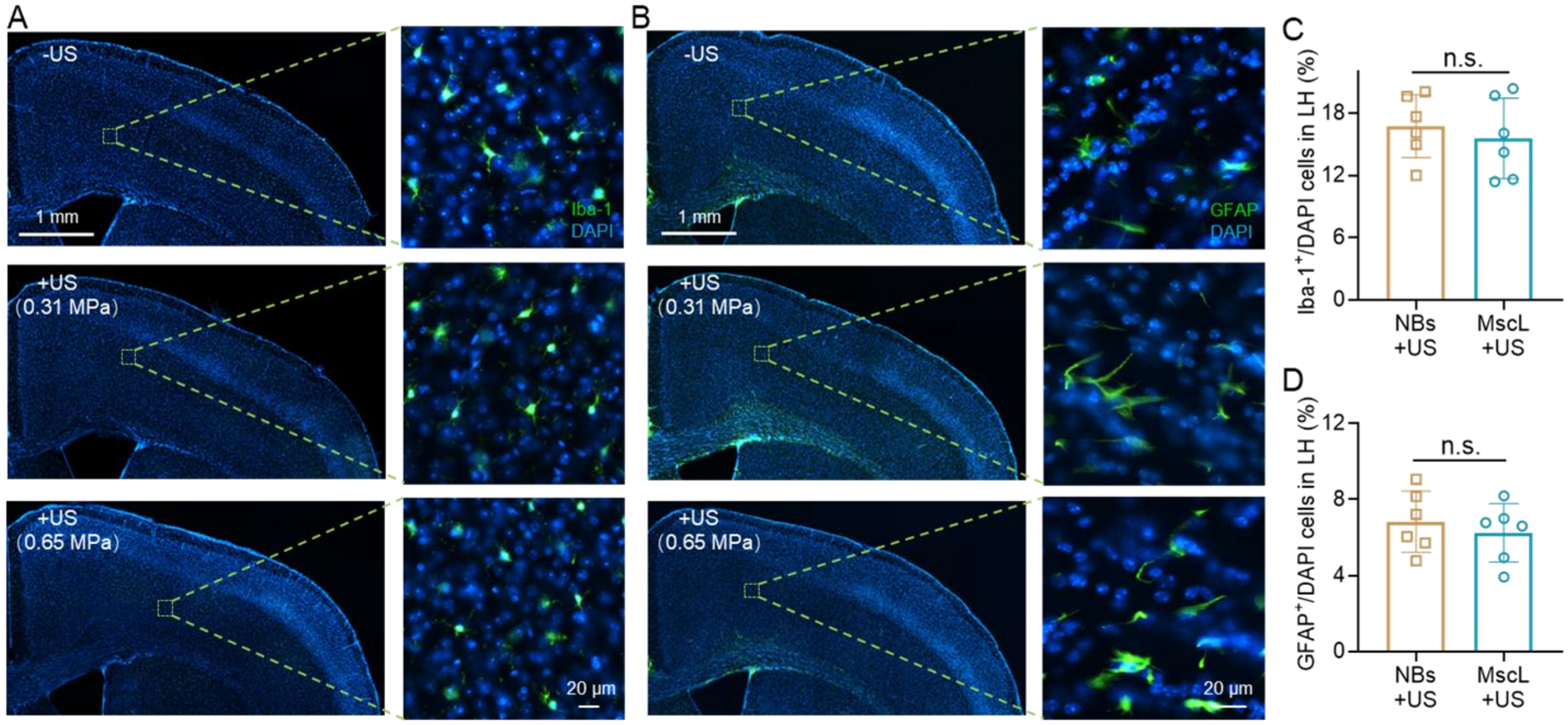

Figure S7. Evaluation of different ultrasound stimulation effects on neural inflammation. *Representative images depicting the expression of Iba-1 (**A**) and GFAP (**B**) in mice cortex 24 h after ultrasound stimulation. For both panels, images are representative of 6 independent experiments per condition. Quantification of Iba-1-positive (**C**) and GFAP-positive (**D**) cells as a percentage of DAPI-stained cells in the LH under 0.65 MPa ultrasound stimulation. Error bars indicate mean ± SD (n = 6 mice per group). n.s., not significant (unpaired t test).*

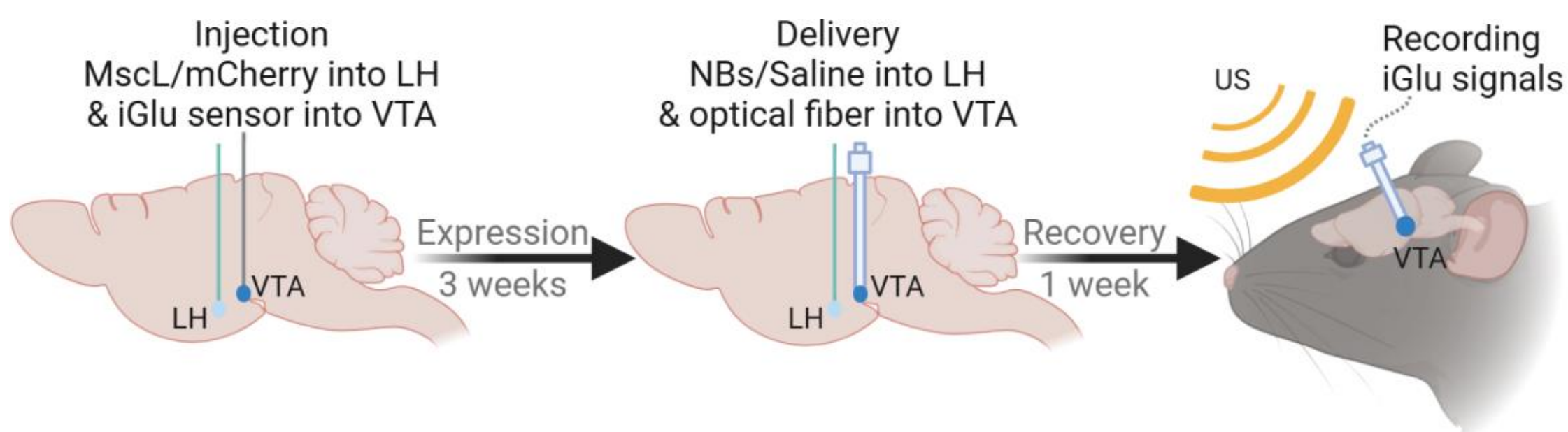

Figure S8. Recording glutamate release in mouse VTA by fiber photometry. *Schematic representation of the experimental process. MscL/mCherry virus was injected into the LH, and the iGlu sensor was injected into the VTA. After three weeks of virus expression, NBs/saline were delivered, and an optical fiber was embedded. One week later, glutamate release was recorded by fiber photometry during US stimulation.*

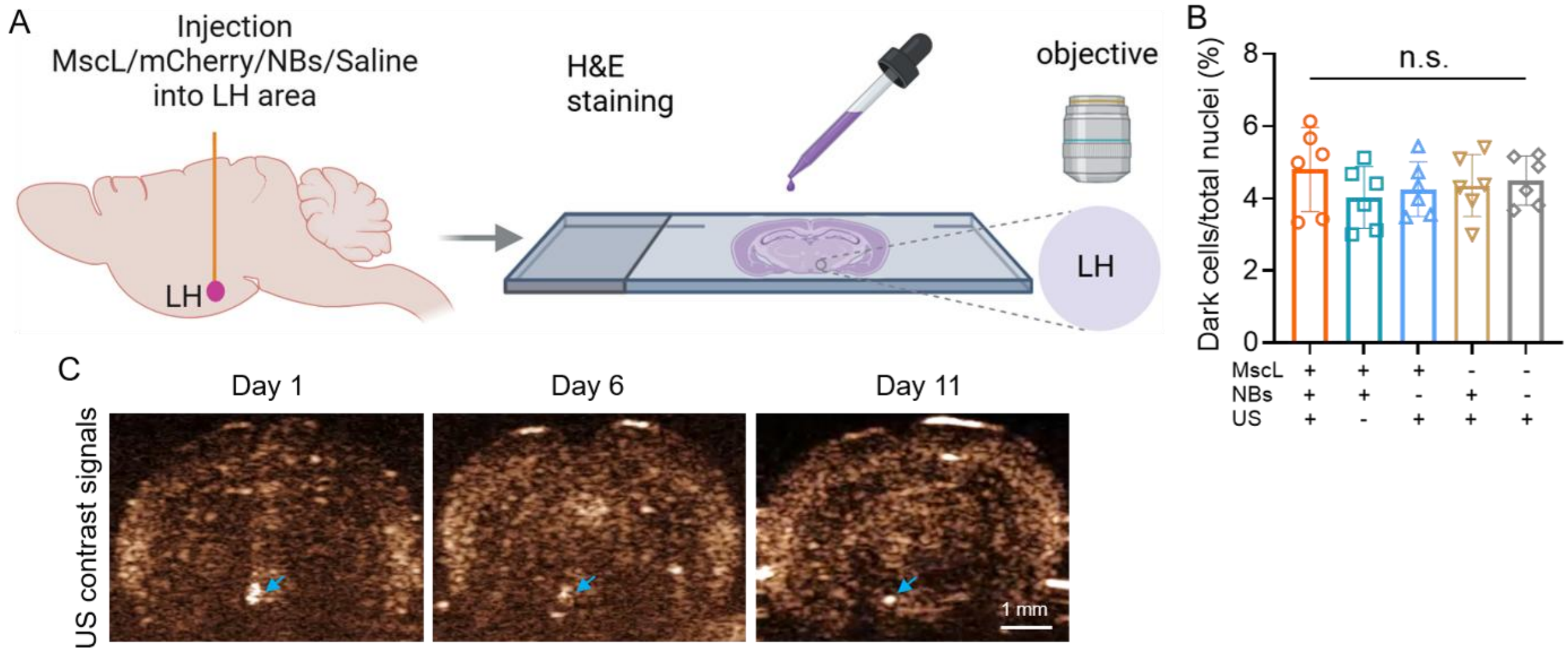

Figure S9. H&E staining and NBs stability assessment. *(**A**) Schematic depiction of the experimental process. (**B**) Quantification of dark-stained cells in H&E-stained sections across different treatment groups. Error bars indicate mean ± SD. Statistical analysis showed no significant differences (n.s.) as determined by one-way ANOVA. (**C**) Contrast-mode ultrasound images of NBs-injected mice brains at multiple time points post-injection.*

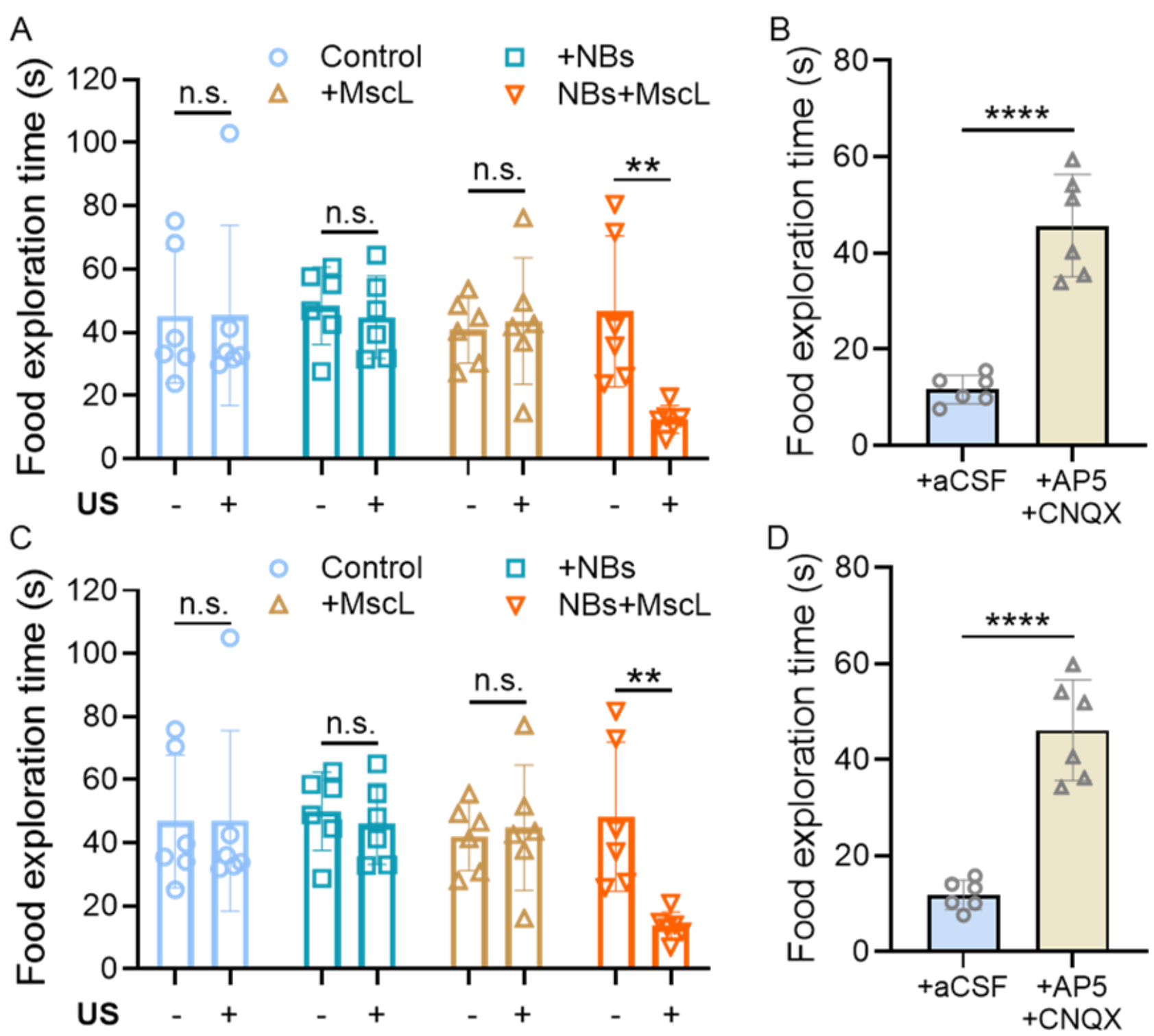

Figure S10. Food exploration time. *Time spent in the food-proximate region across treatment conditions, quantified using two distances (2.0 cm in **A**, **B**; 2.5 cm in **C**, **D**). n = 6 mice per group. Error bars indicate mean ± SD. n.s., not significant; **p < 0.01; ****p < 0.0001 (two-way ANOVA for panels **A** and **C**; unpaired t-test for panels **B** and **D**).*
